# The gut microbiota contributes to changes in the host immune response induced by *Trichinella spiralis*

**DOI:** 10.1371/journal.pntd.0011479

**Published:** 2023-08-16

**Authors:** Chengyao Li, Yi Liu, Xiaolei Liu, Xue Bai, Xuemin Jin, Fengyan Xu, Hong Chen, Yuanyuan Zhang, Isabelle Vallee, Mingyuan Liu, Yong Yang

**Affiliations:** 1 State Key Laboratory for Zoonotic Diseases, Key Laboratory for Zoonosis Research of the Ministry of Education, Institute of Zoonosis, College of Veterinary Medicine, Jilin University, Changchun, China; 2 UMR BIPAR, Anses, Ecole Nationale Vétérinaire d’Alfort, INRA, University Paris-Est, Animal Health Laboratory, Maisons-Alfort, France; 3 Jiangsu Co-innovation Center for Prevention and Control of Important Animal Infectious Diseases and Zoonoses, Yangzhou, Jiangsu, China; 4 School of Basic Medical Science, Shan Xi Medical University, Taiyuan, China; University of Liverpool, UNITED KINGDOM

## Abstract

The gut microbiota plays an important role in parasite-host interactions and the induction of immune defense responses. *Trichinella spiralis* is an important zoonotic parasite that can directly or indirectly interact with the host in the gut. Changes in the gut microbiota following infection with *T*. *spiralis* and the role of the gut microbiota in host immune defense against *T*. *spiralis* infection were investigated in our study. 16S rRNA sequencing analysis revealed that infection with *T*. *spiralis* can reduce the diversity of the gut microbiota and alter the structure of the gut microbiota during early infection, which was restored when the worm left the gut. Antibiotic treatment (ABX) and fecal bacterial transplantation (FMT) were used to investigate the role of the gut microbiota in the host expulsion response during infection with *T*. *spiralis*. We found that ABX mice had a higher burden of parasites, and the burden of parasites decreased after fecal bacterial transplantation. The results of flow cytometry and qPCR revealed that the disturbance of the gut microbiota affects the proportion of CD4+ T cells and the production of IL-4, which weakens Th2 responses and makes expulsion difficult. In addition, as the inflammatory response decreased with the changes of the microbiota, the Th1 response also decreased. The metabolomic results were in good agreement with these findings, as the levels of inflammatory metabolites such as ceramides were reduced in the ABX group. In general, *T*. *spiralis* infection can cause changes in the gut microbiota, and the presence or absence of microbes may also weaken intestinal inflammation and the expulsion of *T*. *spiralis* by affecting the immune response of the host.

## Author summary

Due to the complexity of intestinal environment and function, we are interested in the relationship between *Trichinella spiralis* and the gut microbiota. In our study, we reported the effects of *T*. *spiralis* infection on the gut microbiota of the host and gut microbiota intervention in *T*. *spiralis* expulsion by influencing the immune status of the host. On the one hand, we demonstrated that the infection of *T*. *spiralis* in the intestinal stage could lead to a decrease in the diversity of the gut microbiota and changes in community structure, and these results are conducive to *T*. *spiralis* invasion. On the other hand, the gut microbiota plays an important role in *T*. *spiralis* infection. The balance of gut microbiota is largely involved in the invasion and colonization of *T*. *spiralis* and the state of host immunity. Our findings further elucidated the relationship among host, parasite and gut microbiota, and provided new insights into the prevention and control of *T*. *spiralis*. In addition, the study also provides a new basis for the important role of the gut microbiota in pathogen infection and host homeostasis.

## 1 Introduction

A large number of bacteria that are present in the intestine of mammals are attached to the surface of the intestinal mucosa, and they participate in host digestion, metabolism, immune regulation, energy conversion, mucosal development, barrier maintenance and other physiological functions [1–3]. In general, the gut microbiota lives in a relatively stable environment in dynamic equilibrium, and its composition and function have important impacts on the health and homeostasis of the host [4]. Changes in the community, abundance, proportion and location of the gut microbiome lead to impaired barrier function, which leads to pathological changes in the host [5].

In addition to the gut microbiota, there are some parasites that can colonize the intestine, including protozoa and nematodes, which share the intestinal microenvironment with the gut microbiota during intestinal colonization [6,7]. Studies have shown that this tripartite partnership (host-worm-bacteria) has led to complex adaptations that have and will continue to shape the host’s physiology.

*Trichinella spiralis* is a nematode parasite. It has the ability to infect a variety of mammals, including pigs, horses, reptiles and birds, but only causes disease in humans [8,9]. Trichinosis is an important foodborne zoonotic disease, and hosts are mostly infected by ingesting uncooked or semi-cooked meat containing *T*. *spiralis* larvae [10]. After digestion, the infective muscle larvae (ML) then enter the epithelium of the intestine, where they develop into adult worms (AD) after molting four times and temporarily reside in the intestine, where the AD mate and release newborn larvae (NBL) that enter the host via the blood. Eventually, they migrate to muscle tissue to grow and settle [11,12]. Previous studies have reported that *T*. *spiralis* interacts with the host through its own mechanical movement or via secreted products with functional molecules [13–15]. However, parasites and microbes in the intestine are like "old friends" and have evolved with the host for longtime [16]. The interaction among worms, bacteria and their mammalian hosts affect not only the host-worm and host-microbiome interactions but also the relationship between worms and the microbiome [17].

In recent years, specific gut microbiota patterns have been shown to be associated with the colonization of common parasites. With advances in sequencing technology, studies investigating the host microbiota after parasite infection with 16S rRNA sequencing technology have also become increasingly abundant [18,19]. Although changes in the gut microbiota after infection with *T*. *spiralis* have been reported [20], there are few studies using 16S rRNA sequencing technology, and research on different infection periods is incomplete [21]. In addition to the impact of *T*. *spiralis* infection on the composition of the gut microbiota, whether the microbiota is involved in the parasitism and expulsion of *T*. *spiralis* in the host is a key area of concern. It has been reported that ovum hatching and establishment of *Trichuris muris* in mice require the presence of the microbiota [22]. Similarly, the gut microbiota appears to be critical for *Heligmosomoides polygyrus* to thrive [23]. However, the role and mechanism of the gut microbiota in colonization and expulsion of *T*. *spiralis* have not yet been elucidated. Here, we revealed the composition of and changes in the host microbiome at various stages of *T*. *spiralis* infection and explored the impact of the gut microbiota on expulsion and related host immune mechanisms. These results advance our understanding of worm-host interactions and the putative mechanisms by which this or other worms influence mucosal immunity and disease susceptibility.

## 2 Materials and methods

### Ethics statement

All mice were handled strictly in accordance with the Animal Ethics Procedures and Guidelines of the People’s Republic of China. The protocol was approved by the Institutional Animal Care and Use Committee of Jilin University (Protocol # 20170318).

### Mice, parasites, and sample collection

Male C57BL/6 mice (6 weeks) and female Wistar rats (8 weeks) were purchased from the Experimental Animal Center of Jilin University. *T*. *spiralis* (ISS 534 strain) was maintained in female Wistar rats, and ML were recovered from the muscles of infected mice using a modified pepsin–hydrochloric acid digestion method as previously described [24]. To make the gut microbiota background as consistent as possible before the experiment began, 6-week-old C57BL/6 mice were cohoused in a large double layer wire mesh cage (70 cm × 100 cm × 50 cm) within seven standard mouse cages (350 mm × 230 mm × 160 mm) for 2 weeks. Each standard cage contained five mice, all of which were given the same feed, water, bedding, and environment. Subsequently, 35 mice were divided into a control group (n = 11) and an infection group (n = 24). The infection group was orally administered 300 ML that were treated aseptically in 500 μL saline, and the same amount of saline was given to the control group. In the control group, six mice were sacrificed on day 0, and five mice were sacrificed on day 40. In the infected group, six mice were sacrificed on days 3, 8, 17, and 40. Accordingly, we named these groups CK, CK40, Ts3, Ts8, Ts17, and Ts40. The mice were euthanized via CO_2_ emission, and the eyeball was exophthalmia by keeping and blood was collected from the retro-orbital sinus/plexus [25], and serum samples were obtained by centrifugation at 4°C and 1,144 g for 15 min. The colon contents of the mice were collected under aseptic conditions. The colon contents were immediately placed in a sterile centrifuge tube, frozen using liquid nitrogen, and then transferred to -80°C for storage. Similarly, the same collection and storage methods were used for colon contents from the control group, no-antibiotic treatment (ABX) group (8 days post infection(dpi)), and ABX group (8 dpi) for metabolome analysis.

### Antibiotic treatment (ABX)

Mice were treated with a cocktail of antibiotics (ampicillin 1 g/L; neomycin sulfate 1 g/L; metronidazole 1 g/L; streptomycin sulfate 1 g/L; and vancomycin 0.5 g/L) in the drinking water for 2 weeks. All these antibiotics were purchased from Sigma (Sigma, St.Louis, MO, USA).

### Counts of adult worm (AD), muscle larvae (ML) and new born larvae (NBL) of *T*. *spiralis*

AD and ML were collected as described previously [24,26]. The AD collected in the small intestine of each mouse were counted. The calculation of ML is expressed as the larvae burden per gram of body weight, also known as LPG. For NBL, a single female adult on 6 dpi was selected and cultured for 24 h, and the amount of production was counted under an inverted microscope (Olympus, Tokyo, Japan).

### ELISA

Antibodies were detected by ELISA. Parasite-specific sIgA, IgG1, IgG2a and total IgG levels were determined as described previously [27]. For the detection of IgG, IgG1 and IgG2a, *T*. *spiralis* muscle larval homogenate was used as a target antigen (Ag) at 2 μg/mL, and serum was diluted 1/50. For the detection of sIgA, 1mg of small intestine tissue was melted at 4°C and the tissue was homogenized thoroughly with a homogenizer in 500 μL PBS. The tissue homogenate was centrifuged at 840 g for 20 min at 4°C, and the supernatant was collected for subsequent use. Mouse IgG1, sIgA, IgG2A and IgG primary antibodies (Abcam, Cambridge, UK) and HRP-conjugated goat anti-mouse secondary antibodies (Affinity Biosciences, USA) were used for detection. After adding the TME substrate (Tiangen, Beijing, China) and the termination solution (2 M H_2_SO_4_), the absorbance was measured at 450 nm.

### Fecal microbiota transplantation (FMT)

The process of FMT was performed as previously described [28]. Fresh fecal samples were collected from untreated donor mice. Stool pellets were collected from different healthy donors, mixed with sterile water (1 fecal pellet/1 mL sterile water) and homogenized immediately. The sample was centrifuged (100 ×g, 5 min, 4°C), and the supernatant was used for transplantation. The supernatant (200 μL/mouse) was administered by gavage to recipient mice that had been treated with antibiotics for 7 consecutive days.

### FITC-dextran-based detection of intestinal permeability in mice

FITC-dextran (4 kDa, Sigma, St.Louis, MO, USA, FD4) was administered by gavage to each group of mice at 0.6 mg/g in sterile PBS. The mice were euthanized after 4 h, and blood was collected into serum separator tubes. The tube was centrifuged at 9,935 g for 10 min, and the serum was transferred to a new sterile tube and diluted 1:1 with 2% Triton X-100. Serum fluorescence intensity was measured using a Cytation Hybrid Multi-Mode Reader (BioTek, USA) at 485 nm excitation/528 nm emission.

### Flow cytometry

The mesenteric lymph nodes of mice were extracted, ground and filtered through a 70 μm cell sieve. After centrifugation, mesenteric lymph node cells were obtained. Single cell suspension was then stained for flow cytometry with the following antibodies: FITC Hamster Anti-Mouse CD3e (Clone: 145-2C11), PerCP-Cy 5.5 Hamster Anti-Mouse CD3e (Clone:145-2C11), APC Rat anti-Mouse CD4 (Clone:RM4-5), and PE Rat Anti-Mouse CD8a (Clone:53.6–7). All antibodies were purchased from BD (BD Biosciences, New Jersey, USA). Single cells were divided into antibody unlabeled group, single antibody labeled group and multiple antibodies labeled group. Each tube was added to 100 μL PBS buffer, and 10^6^ cells were labeled with 1μg antibodies. The antibodies were incubated for 30 min at 4°C in the dark, and centrifuged at 210 g for 10 min at 4°C to remove the supernatant. After two washes with PBS buffer, a flow cytometer (BD FACS Verse) was used for detection.

### Histopathology

Mice were sacrificed at predetermined times, and the small intestine and diaphragm were collected and fixed in 10% formalin for 24 h. Whole tissue specimens were embedded in paraffin after dehydration. The middle portion of the embedded sample was cut transversely into 4-μm-thick sections and processed for routine hematoxylin and eosin (HE) staining. An HE staining kit (Solarbio, Beijing, China) was used. According to the manufacturer’s instructions, the sections were dewaxed by xylene, rehydrated, and soaked in distilled water for 2 min. After 5 min of hematoxylin staining, unbound hematoxylin was removed with distilled water. Then the cells were differentiated for 10 s, and the sections were quenched 2×5 min with water. The sections were placed in eosin staining solution for 1 min and then dumped into excess liquid for immediate dehydration and fixation sealing. The slides were examined by light microscopy (Olympus, Tokyo, Japan). For statistical analysis, we used ImageJ (1.53) to measure villus length and crypt depth. First, the Line Selection tool in the ImageJ toolbar was selected. Then a linear selection was created in the image with a known length (according to the scale length). Finally, the Analyze menu was selected, and the Set Scale command was used to set the Known distance to the known length (scale length) and the Unit of length to the unit of measure (μm). A straight line was drawn to the measured position and the “measure” option was used to obtain the value. The experiment was performed by researchers in a blind manner.

### Immunofluorescence assay (IFA)

The experimental procedure was a modification of the original protocol [29]. Briefly, paraffin tissue sections of the small intestine were hydrated with xylene and gradient alcohol and then boiled with EDTA solution (pH = 8.0, Solarbio, Beijing, China) for 15 min for tissue antigen retrieval. After cooling to room temperature, 0.05% trypsin was added for 10 min to destroy the auto-fluorescent fibrous tissue. Then, the sections were blocked with goat serum (Boster, Wuhan, China) for 1 h, and a rabbit claudin-1 antibody (1:200, Affinity Biosciences, USA) was added and incubated overnight at 4°C. The next morning, the sections were returned to room temperature after 2 × 5min PBS (0.5% Tween-200, 1% Triton X-100) washes followed by an additional 5 min PBS wash. A Goat Anti-Rabbit IgG H&L antibody (1:1000, Alexa Fluor 488, Abcam, Cambridge, UK) was incubated with the sections for 45 min at room temperature, and the above washing procedure was repeated. Subsequently, DAPI (Invitrogen) was used for counterstaining of nuclei. Finally, an anti-fluorescence quenching agent (Invitrogen) was used for mounting and scanning using a laser confocal microscope (Olympus Fluo view 1000, Tokyo, Japan). Violet (excitation/emission = 405 nm/455 nm) and blue (excitation/emission = 488 nm/515 nm) lasers were used for observation.

### qRT–PCR

Total RNA from the small intestine was extracted by using TRIzol as previously described [30]. The RNA was reverse transcribed to cDNA according to the manufacturer’s instructions of the *TransScript* One-Step gDNA Removal and cDNA Synthesis Supermix Kit (Trans, Beijing, China). The cDNA was amplified using the SYBR Green qRT–PCR Master Mix Kit (Roche, Switzerland). Mouse genes were amplified using specific primers, and expression was normalized to GAPDH. The experimental results were analyzed using the ΔΔCt method. The primer sequences are shown in Table 1.

**Table 1:** Primer sequences and gene fragment size of amplified genes.

Gene name	Primer sequence (5’-3’)	Product size
*TNF-α*	F: AGCCGATGGGTTGTACCTTG R: ATAGCAAATCGGCTGACGGT	99 bp
*IFN-γ*	F: ATGAACGCTACACACTGCATC R: CCATCCTTTTGCCAGTTCCTC	182 bp
*IL-4*	F: CCACGGATGCAACGACAATC R: AGGACGTTTGGCACATCCAT	102 bp
*IL-13*	F: GGCAGCATGGTATGGAGTGT R: CTTGCGGTTACAGAGGCCAT	132 bp
*MUC2*	F: CCTTAGCCAAGGGCTCGGAA R: GGCCCGAGAGTAGACCTTGG	368 bp

### High-throughput sequencing of 16S rRNA gene amplicons

Total genomic DNA was extracted using a DNA Extraction Kit (QIAamp 96 PowerFecal QIAcube HT kit, QIAGEN) following the manufacturer’s instructions. The quality and quantity of DNA were verified with a NanoDrop system and an agarose gel. Extracted DNA was diluted to a concentration of 1 ng/μL and stored at -20°C until further processing. The diluted DNA was used as a template for PCR amplification of bacterial 16S rRNA genes with barcoded primers and Takara Gflex DNA Polymerase (Takara Bio, Beijing, China). For bacterial diversity analysis, the V3-V4 variable regions of 16S rRNA genes were amplified with the universal primers 343F and 798R (forward primer, 343F -5’- TACGGRAGGCAGCAG -3’; reverse primer: 798R - 5’- AGGGTATCTAATCCT-3’) (S1 and S2 Tables). Amplicon quality was visualized by gel electrophoresis, and amplicons were purified with AMPure XP beads (Agencourt) and subjected to another round of PCR (S3 and S4 Tables). After purification with AMPure XP beads was performed again, the final amplicons were quantified using a Qubit dsDNA assay kit. Equal amounts of purified amplicons were pooled for subsequent sequencing.

Sequencing was performed on an IIIumina NovaSeq 6000 (IIIumina Inc., San Diego, CA; OE Biotech Company; Shanghai, China) with 250 bp paired-end reads. Trimmomatic (0.35) [31] was used to scan low-quality raw data sequences using the sliding window method. When the mass was lower than 20, the sliding window with an average base quality that was lower than the threshold value was cut out, and the sequences that had a length of less than 50 bp were removed. Second, Flash (1.2.11) [32] was used to splice the qualified double-ended raw data generated from the previous step. The maximum overlap during sequence splicing was 200 bp, and a complete paired end sequence was obtained. Split library software in QIIME (1.8.0) [33] was used to remove sequences with N bases in the paired-end sequence, sequences with a single base repetition greater than 8, and sequences with lengths less than 200 bp to obtain clean tag sequences. Then, Flash (1.2.11) was used to remove the chimeras in the clean tags, and valid tags were obtained for subsequent OTU partitioning. Finally, quality control of the whole process was statistically performed before the following bioinformatics analysis.

### Immunohistochemistry and periodic acid–Schiff (PAS) staining

Small intestinal tissue sections were deparaffinized and subsequently boiled in EDTA buffer (pH 8.0) for 15 min to improve antigen retrieval. Then, the tissue sections were treated with 3% hydrogen peroxide solution to block endogenous peroxidase activity. Nonspecific binding was blocked by incubation with blocking serum for 30 min at room temperature. Subsequently, the sections were incubated with an anti-MUC2 rabbit polyclonal antibody (1:400 dilution; Abcam, Cambridge, UK) overnight at 4°C. The sections were then washed with PBS and treated with a peroxidase-conjugated anti-rabbit secondary antibody (Beijing Biosynthesis Biotechnology Co., Ltd., China) for 30 min at room temperature, followed by 3,30-diaminobenzidine (DAB) and hematoxylin staining. Images were captured using an Olympus BX53 fluorescence microscope (Olympus, Tokyo, Japan). For PAS staining, deparaffinized sections were washed with water and then subjected to Alcian blue staining, oxidant treatment, Schiff staining, hematoxylin staining, acid differentiation treatment, and Scott staining in sequence. Finally, the sections were dehydrated with ethanol, cleared with xylene, and then mounted for observation (Solarbio, Beijing, China). The slides were examined by light microscopy (Olympus, Tokyo, Japan). Researchers were blinded to the groups and counted the number of brown/ deep purple particles on single crypt, which were considered goblet cells, in three experiments.

### Metabolomic analysis

Metabolome extraction, purification, and derivatization were performed in a single process according to the manufacturer’s instructions. 30 mg of sample was added to a 1.5 mL Eppendorf tube, with 20 μL of Lyso PC 17:0 (0.1 mg/mL) dissolved in methanol as an internal standard, and then 300 μL methanol: water (1/1, vol/vol) solution was added. Two small steel balls were added to each tube, and the mixed was pre-cooled at -20°C for 2 min, and then added to a grinder (60 Hz, 2 min). Then, 300 μL of chloroform was added to each sample, and the mixtures were vortexed for 30 s, extracted by ultrasonication for 10 min in ice-water bat, then placed at -20°C for 20 min, and centrifuged at 4°C (13,443 g) for 10 min before 200 μL of the chloroform layer was decanted into sample vials. Then, 300 μL chloroform: methanol (2/1, vol/vol, with 0.1mM BHT) solution was added to the residue of the samples, and the sample was vortexed for 30 s, extracted by ultrasonication for 10 min in ice-water bath, placed at -20°C for 20 min, and then centrifuged at 4°C (15,777 g) for 10 min prior to decanting the chloroform layer to sample vials. The two subnatant fractions were combined and mixed well. Then, the mixed subnatant (100 μL) was dried under a nitrogen stream and re-dissolved in 200 μL of an isopropanol: methanol (1/1, vol/vol) solution, the mixture was vortexed for 30 s and extraction was performed by ultrasonication for 3 min in an ice-water bath. The solution was transferred to a 1.5 mL centrifuge tube and incubated for 2 h at -20°C. After centrifugation for 10 min (13,443 g, 4°C), 150 μL supernatant was taken and placed into a liquid chromatography-mass spectrometry (LC-MS) injection vial with a linear tube for subsequent ultra-performance liquid chromatography–tandem mass spectrometry (UPLC-MS/MS) analysis. Quality control (QC) samples were prepared by mixing aliquots of the sample to generate a pooled sample. All chemicals and solvents were analytical or high-performance liquid chromatography (HPLC) grade. Water, methanol, acetonitrile, isopropanol, and formic acid were purchased from Thermo Fisher Scientific (Thermo Fisher Scientific, Waltham, MA, USA). Ammonium formate, and chloroform were obtained from Titan Chemical Reagent Co. Ltd (Shanghai, China). Lyso PC 17:0 was purchased from Avanti.

Detection was performed using heated electrospray ionization (HESI) in positive and negative ion modes. Samples were separated by a Dionex U3000 UHPLC (Thermo Fisher Scientific) and analyzed by mass spectrometry using a Q Exactive Plus (Thermo Fisher Scientific). This part of the experiment was entrusted to Shanghai OE Biological Co., Ltd.

### Bioinformatic analysis

For 16S rRNA sequencing, clean reads were subjected to primer sequence removal and clustering to generate OTUs using Vsearch software (2.4.2) with a 97% similarity cutoff [34]. The representative read of each OTU was selected using the QIIME package (1.8.0). All representative reads were annotated with Silva database Version 123 (16S rRNA) using the RDP classifier (the confidence threshold was 70%) [35].

For the analysis of lipid metabolism, the original Q Exactive LC-MS/MS data in raw format were processed by the software Lipid Search to determine the MS^n^ and the exact mass-to-charge ratio (m/z) of parent ions. The molecular structures of lipids and the additive mode of their positive and negative ions were identified according to the parent ions and multi-stage mass spectrometry data obtained from each sample. The results were aligned according to a specific retention time ranges and combined into a single report to organize the original data matrix. All peak signals from each sample were normalized. The extracted data were then further processed by removing any peaks with missing data in more than 50% of the groups and by replacing zeros with half of the minimum value. A data matrix was combined from the positive and negative ion data. The matrix was imported into R to carry out Principal Component Analysis (PCA) to observe the overall distribution among the samples and the stability of the whole analysis process. Orthogonal Partial Least-Squares-Discriminant Analysis (OPLS-DA) and Partial Least-Squares-Discriminant Analysis (PLS-DA) were utilized to distinguish the metabolites that differed between groups. To prevent overfitting, 7-fold cross-validation and 200 Response Testing (RPT) were used to evaluate the quality of the model. Variable Importance of Projection (VIP) values obtained from the OPLS-DA model were used to rank the overall contribution of each variable to group discrimination. A two-tailed Student’s T -test was further used to verify whether the metabolites of difference between groups were significant. Differential metabolites were selected as metabolites with levels resulting in VIP values greater than 1.0 and differences with *p* values of less than 0.05.

### Statistical analysis

**Fig 1:**
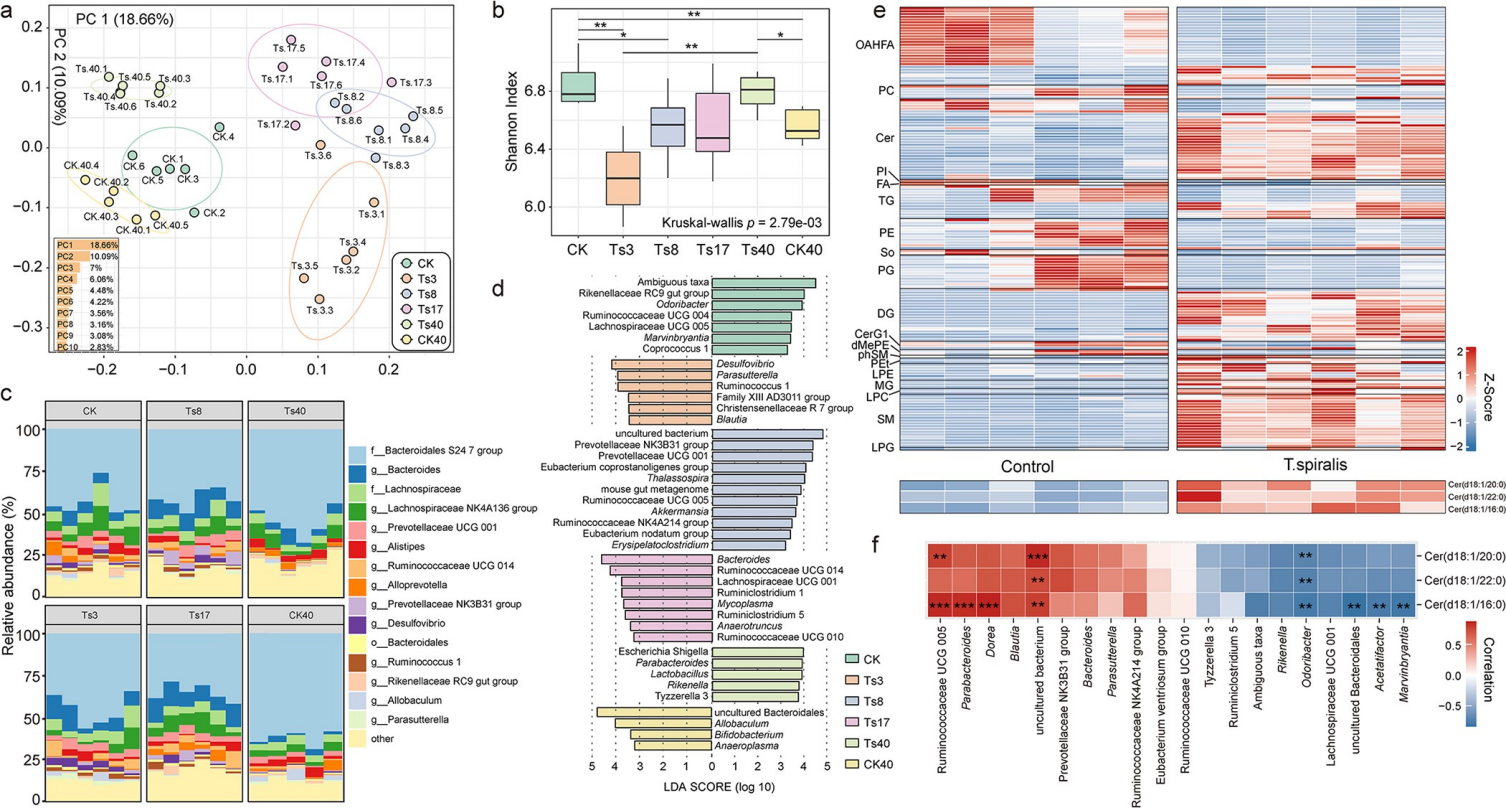
Changes in the gut microbiota and metabolites after *T*. *spiralis* infection. **(a)** The PCOA analysis based on unweighted UniFrac distance. PC1 and PC2 are the two main coordinates that explained the largest proportion of the variance between samples. The color indicates the grouping, a point indicates a sample, and similar samples are clustered together. The bottom left corner of the image shows the ratio of PC1-10 in the bar graph. **(b)** The distribution of the Shannon index in each group and the *p* value of the difference in the diversity index between groups. **(c)** Scale histogram based on relative abundance at the genus level. The proportion of TOP15 genera according to the relative abundance in different groups is shown. Each column represents an individual mouse. **(d)** LFfSe analysis illustrating the different genera in each group. The effect size threshold of LDA was set to 3.0. **(e)** Heatmap clustered according to different classes of metabolites of colon contents showing the standardized lipid metabolites content in the *T*. *spiralis* (TS8) and control group. OAHFA: (O-acyl)-hydroxy fatty acids, PC: phosphatidylcholine, Cer: ceramide, PI: phosphatidylinositol, FA: fatty acids, TG: triacylglycerol, PE: phosphatidylethanolamine, So: sphingoshine, PG: phosphatidylglycerol, DG: diacylglycerol, CerG1: glycosylceramide, dMePE: dimethyl-phosphatidyl ethanolamine, phSM: phytosphingosine, PEt: phosphatidylethanol, LPE: lysophosphatidyl ethanolamine, MG: monoglyceride, LPC: lysophosphatidylcholine, SM: sphingomyelin, LPG: lysophosphatidylglycerol. The contents of Cer (d18:1/20:0), Cer (d18:1/22:0) and Cer (d18:1/16:0) in the two groups are shown at the bottom of the image. Each column represents an individual mouse. **(f)** Spearman correlation coefficient between metabolites and gut microbiota. Blue indicates a negative correlation, and red indicates a positive correlation. All statistical differences are expressed as* p<0.05, ** p<0.01, ***p<0.001.

As showed in Fig 1, the R (v.4.0) platform was used for statistical analysis and *ggpolt2* was used for data visualization (**S1 Code**). The *GUniFrac* package was used to calculate the UniFrac distance. The Shannon index was calculated by the *vegan* package. The Wilcoxon signed-rank test and Kruskal-Walli’s test were used to assess the differences between each group and the control group. The analysis of Fig 1D was carried out on the website (https://huttenhower.sph.harvard.edu/galaxy/). The *psych* package was used to calculate the Spearman correlation coefficient between the microbiota and the metabolites. In addition, other statistical analyses and graph generation were performed using GraphPad Prism 9 (9.0.0) with one-way or two-way ANOVA followed by Tukey’s multiple comparison post-test. For all results, *p* values are indicated as *p < 0.05, **p < 0.01, or ***p < 0.001, and ****p < 0.0001.

## Results

### 3.1 *T*. *spiralis* infection changes the diversity of the gut microbiota in mice

To investigate the effect of *T*. *spiralis* infection on the gut microbiota of the host, a total of 35 samples from the colon were collected for 16S rRNA sequencing. Finally, the number of clean reads obtained after quality control ranged from 12910~26266. The average length of valid reads of each sample ranged from 426.67~433.67 bp, and the number of OTUs ranged from 444~738 **(S1A Fig)**. The OTU rarefaction curve also indicated that the sampling work had sufficient sequences to analyze bacterial diversity **(S1B Fig)**.

To exhibit the difference in gut bacterial composition between infected groups and control groups (CK and CK40), the β diversity of the gut microbiota was evaluated first. PCOA based on unweighted UniFrac distances showed that the control groups could not be separated (**Fig 1A**). However, Ts3, Ts8, and Ts17 were separated from the control groups by PC2 (explaining 10.09% of the variance), while Ts3, Ts8, Ts17 and Ts40 were separated from the control groups by PC1 (explaining 18.66% of the variance). Notably, the differences among the Ts3, Ts8 and control groups were particularly pronounced. Compared with the control, the Ts3 dpi group had a lower Shannon index, which indicated a lower community diversity. As time progressed, the α diversity gradually recovered and the mean diversity at 8–17 dpi was comparable to that of the control group at day 40, and at 40 dpi, it reached a level comparable to that of the control group at day 0 (**Fig 1B**). In short, this result suggests that early infection with *T*. *spiralis* significantly affects the composition of the gut microbiota.

Next, to clarify differences in relative abundance at different taxonomic levels, we investigated the community composition. At the family level, the Ts3 groups showed significantly higher abundance of Alcaligenaceae and Rhodospirillaceae than the CK group, and the relative abundance of Porphyromonadaceae was lower. Rhodospirillaceae and Bacteroidaceae abundance increased in the Ts8 group; in contrast, Rikenellaceae abundance decreased. However, in Ts40, Lactobacillaceae, a common probiotic, showed significantly increased abundance **(S1C Fig)**.

At the genus level, the bacteria with a relatively high abundance in the CK group were mainly those related to the production of short-chain fatty acids, such as *Lachnospiraceae NK4A136 group*, *Prevotellaceae UCG 001*, *Alistipes*, *Allobaculum* and *Parasutterella*. However, it was found that the abundance of *Parasutterella* and *Desulfovibrio* was significantly increased during the intestinal phase of infection (**Fig 1C**). When we performed LEfSe (LDA Effect Size) analysis of each group, we found that some beneficial bacteria, such as *Rikenellaceae RC9 gut group*, *Odoribacter*, *Ruminococcaceae UCG 004* and *Lachnospiraceae UCG 005*, was more abundant in the control group. However, the dominant genera in the Ts3 group were some inflammation-related bacteria, such as *Desulfovibrio*, and *Family XIII AD3011 group* [36,37]. In addition, in Ts8 group, the dominant bacteria included harmful bacteria such as *Ruminococcaceae NK4A214 group* and *Erysipelatoclostridium*, and beneficial bacteria such as *Prevotellaceae NK3B31 group* and *Prevotellaceae UCG 001*. Subsequently, in the Ts17 group, the microbiota gradually recovered, and *Ruminococcaceae UCG 014* and *Lachnospiraceae UCG 001* accounted for the majority of bacteria. In contrast to the increased abundance of inflammation-related microbiota during the intestinal phase, multiple probiotics, including *Lactobacillus*, exhibited increased abundance in the Ts40 group (**Fig 1D**). This suggests that infection with *T*. *spiralis* for different periods perturbed the gut community composition, which may be closely related to parasitism.

Based on the life cycle of *T*. *spiralis* and the altered gut microbiota caused by infection in the above study, we further analyzed metabolites from colon contents on day 8 of infection. The results showed that 92 metabolites had increased levels and 67 decreased levels in infected animals than in the uninfected group. The metabolites with decreased levels included fatty acids (FA), O-acyl-omega-hydroxy fatty acids (OAHFA), phosphatidylethanolamine (PE) and phosphatidylglycerol (PG), while sphingomyelin (SM) and some harmful ceramide products were among the metabolites with increased levels **(Fig 1E and S5 Table)**. Subsequently, the correlations of these metabolites with the gut microbiota were investigated, and it was found that the levels of the three major ceramides in the infection group were positively correlated with Prevotellaceae and Ruminococcus abundance and significantly negatively correlated with the abundance of Odoribacteraceae **(Fig 1F)**, which was reported to be able to produce secondary bile acids with good anti-inflammatory and antibacterial activity [38]. Therefore, we suggest that the initial inflammatory state of the gut after *T*. *spiralis* infection is not only due to the direct interaction between *T*. *spiralis* and the host but also the decrease in the levels of bacteria that produce anti-inflammatory metabolites and the increase in the levels of bacteria that produce inflammatory substances such as ceramides. However, whether the presence of the microbiota is involved in host intestinal inflammation and deworming remains unknown.

### 3.2 Intestinal inflammation during *T*. *spiralis* infection is related to the gut microbiota

**Fig 2:**
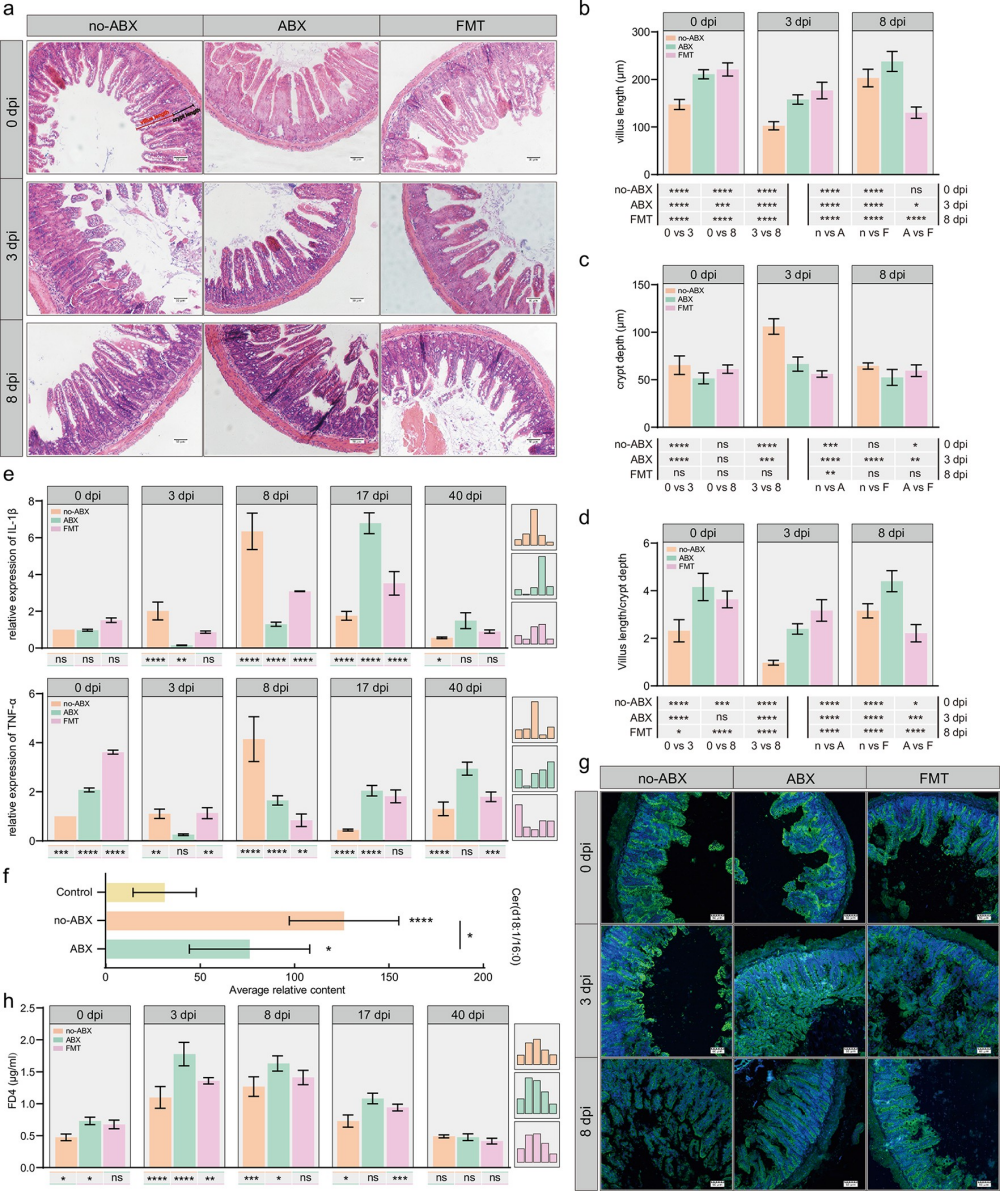
The gut microbiota is involved in intestinal homeostasis. **(a)** Representative HE-stained sections of small intestines from different groups (no-ABX, ABX, FMT) at specific time points (0 dpi, 3 dpi, 8 dpi) (200 ×, scale bar 50 μm). The range of measurements of crypt depth and villus length is also shown. **(b-d)** From the HE-stained sections, villus length **(b)**, crypt depth **(c)**, and villus length/crypt depth **(d)** were determined (n = 3, 8 villus/crypts per mouse, data the represent mean ± SD, two-way ANOVA with Tukey’s post-test). **(e)** The relative expression of TNF-α and IL-1β was detected by qPCR at specific time points (0 dpi, 3 dpi, 8 dpi, 17 dpi, 40 dpi). mRNA expression of different groups (normalized to GAPDH) relative to the 0 dpi-no-ABX group (n = 3, data represent the mean ± SD, two-way ANOVA with Tukey’s post-test). **(f)** The levels of ceramide in the ABX group and no-ABX group at 8 dpi and the control group at day 8 (n = 6, data represent the mean ± SD, two-way ANOVA with Tukey’s post-test). **(g)** Representative images of immunofluorescence for the localization and expression of the tight junction protein Claudin-1 in the small intestine of different groups at specific time points (0 dpi, 3 dpi, 8 dpi). Claudin-1 expression is showed in green (Alexa Fluor-488), and nuclei are showed in blue (DAPI) (scale bar 60 μm). **(h)** FD4 concentration in the serum from different groups at specific time points (0 dpi, 3 dpi, 8 dpi, 17 dpi, 40 dpi) normalized to the 0 dpi-no-ABX group (n = 3, data represent the mean ± SD, two-way ANOVA with Tukey’s post-test). FD4 was used to study intestinal permeability. All statistical differences are expressed as ns = no significant difference, *p<0.05, **p<0.01, ***p<0.001, ****p<0.0001.

To explore the role of the gut microbiota in intestinal inflammation caused by *T*. *spiralis*, we treated C57BL/6 mice with a cocktail of antibiotics administered in the drinking water and then subjected them to FMT **(S2 Fig)**. The 16S rRNA sequencing results showed that ABX could eliminate most of the gut bacteria, as the Shannon index in the ABX group was significantly lower than that in the no-ABX group (**S3 Fig**). At the same time, the intestinal pathology of the two groups was also compared, and it was found that there was a significant difference between the ABX group and the no-ABX group before infection (**Fig 2A**). However, *T*. *spiralis* infection led to the presence of short villi and hyperplasia of crypts at 3 dpi, the levels of which increased at 8 dpi. Villus length changes in the ABX and no-ABX groups were basically the same, but crypt hyperplasia was not as obvious in the ABX groups, and intestinal inflammation was not alleviated or even increased at 3 dpi in the FMT group (**Fig 2A–2D**). Subsequently, we determined the expression of TNF-α and IL-1β, the representative inflammatory factors in the small intestine during the early stage of infection by qPCR, and the results were consistent with the pathological results of the sections. *T*. *spiralis* infection caused significant intestinal inflammation, and the expression of inflammatory markers in the ABX group was lower than that of the no-ABX group at 3 and 8 dpi, which was reversed at 17 and 40 dpi. However, the FMT group showed partially recovery of the ABX-induced decrease in the levels of inflammatory cytokines **(Fig 2E)**. In addition, we found that the ABX group produced fewer ceramides than the untreated group **(Fig 2F)**. Therefore, we speculate that the increased intestinal inflammation that occurs after infection with *T*. *spiralis* is related to the gut microbiota and its metabolites. However, when we evaluated the intestinal permeability after infection and measured the expression of tight-junction protein in intestinal sections, it was found that the ABX group even had higher permeability than the no-ABX group after infection, which was inconsistent with the above results, and whether the bacterial community was also involved in other development of *T*. *spiralis* needs further investigation **(Fig 2G and 2H)**.

### 3.3 The expulsion of *T*. *spiralis* in mice depends on the gut microbiota

**Fig 3:**
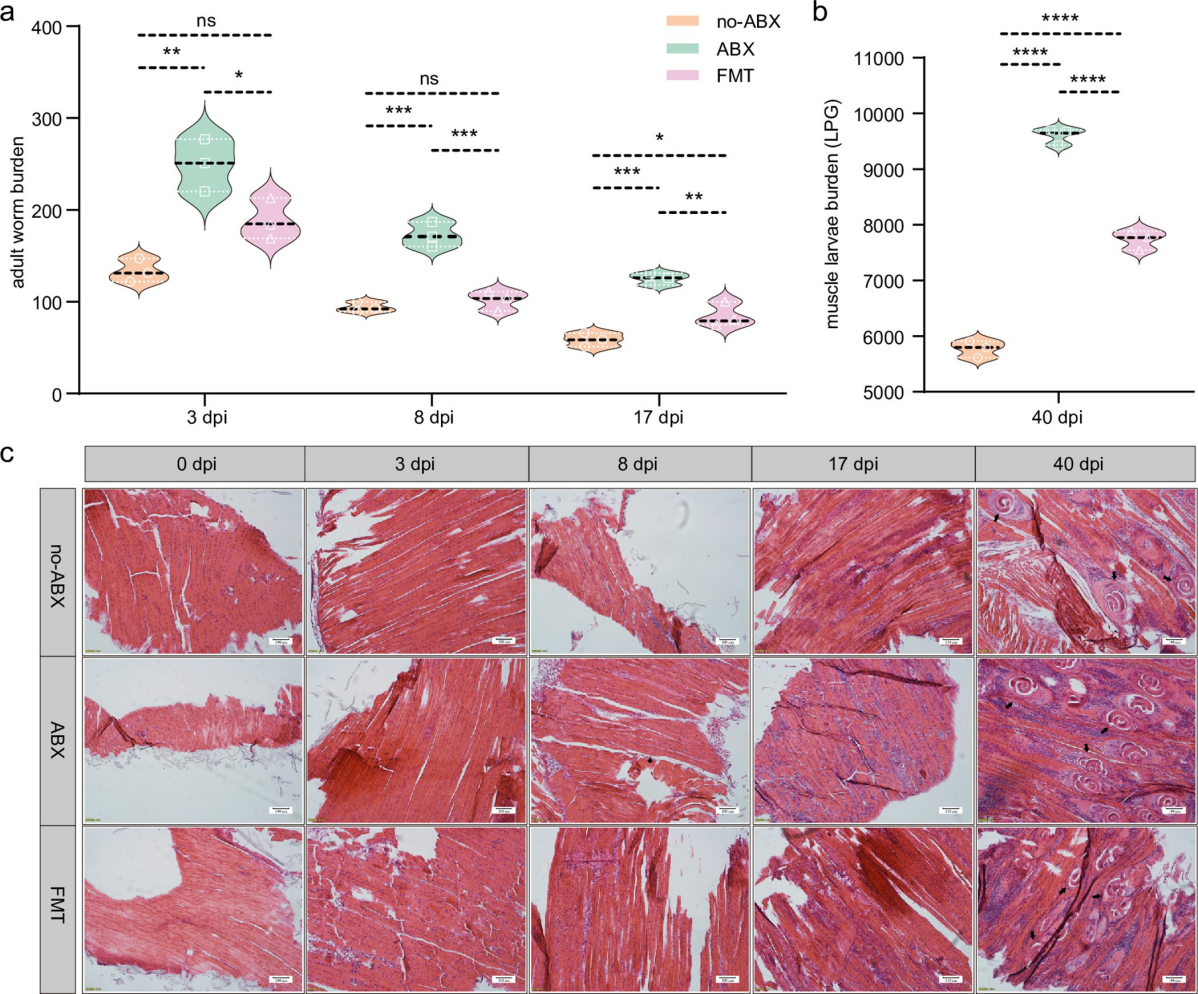
The gut microbiota is involved in the expulsion of *T*. *spiralis*. **(a)** Worms were counted after washing of the small intestine at 0 dpi, 3 dpi, 8 dpi and 17 dpi in different groups (no-ABX, ABX, FMT). **(b)** Muscle larvae (ML) were counted in the muscle at 40 dpi in different groups. LPG is used to represent the number of muscle larvae per gram. (n = 3, data represent the mean ± SD, one-way ANOVA with Tukey’s post-test). All statistical differences are expressed as ns = no significant difference, *p<0.05, **p<0.01, ***p<0.001, ****p<0.0001. **(c)** Representative HE-stained sections of the diaphragm in different groups at specific time points (0 dpi, 3 dpi, 8 dpi, 17 dpi, 40 dpi), *T*. *spiralis* ML are indicated by a black arrow (40 ×, scale bar 100 μm).

Next, we further tested whether the microbiota is involved in the expulsion of *T*. *spiralis* by monitoring the number of AD in the gut at 3, 8, and 17 dpi. The results showed that the burden of parasites in the intestine of ABX mice was nearly twice that of the no-ABX group, with similar results at three different time points **(Fig 3A).** It is worth noting that we cultivated single female AD that were collected at 6 dpi, and we found that there was no significant difference in the number of newborns produced by the AD between the different treatment groups after 24 h **(S4 Fig)**. We also measured the burden of ML and found that the ratio between the two groups was consistent with the results obtained for the AD burden **(Fig 3B)**. To verify that the presence of the gut microbiota can affect parasite infection, we administered the supernatant of fresh fecal suspensions from naive mice to recipient mice ABX. We found that gavage of the microbiota from naive mice (FMT)partially restored the ability to eliminate parasites **(Fig 3A and 3B)**. Consistent with the above results, histopathological evaluation of HE-stained sections showed more muscle inflammation and parasite colonization **(Fig 3C)**. These data indicated that the gut microbiota has an impact on the invasion and colonization, rather than on its reproduction of *T*. *spiralis*. Therefore, we can conclude that the expulsion of *T*. *spiralis* from the host depends on the gut microbiota.

### 3.4 Perturbation of the gut microbiota affects the host’s mucosal immunity

**Fig 4:**
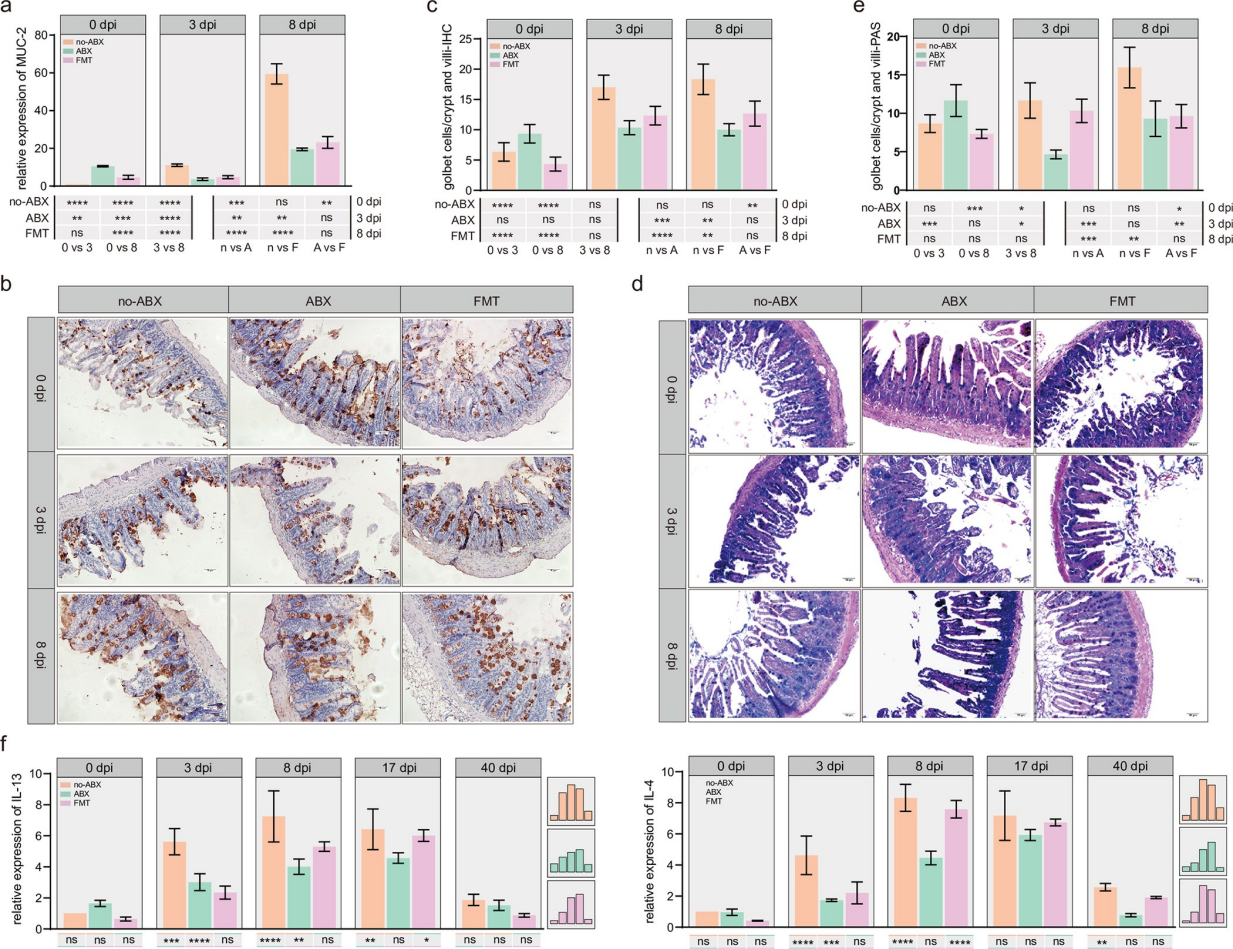
The expulsion of *T*. *spiralis* is affected by the gut microbiota and dependent on the host’s mucosal immunity. **(a)** The relative expression of MUC2 was detected by qPCR. mRNA expression of different groups (normalized to GAPDH) relative to the control 0 dpi-no-ABX group (n = 3, data represent the mean ± SD, two-way ANOVA with Tukey’s post-test). **(b)** Representative images of immunohistochemistry of the duodenum with an anti–MUC2 antibody (200×, scale bar 50 μm), goblet cells are labeled in brown (DAB). **(c)** Goblet cells/crypt were determined (n = 3, 3 crypts per mouse, data represent the mean ± SD, two-way ANOVA with Tukey’s post-test). **(d)** Periodic acid Schiff (PAS) staining of the duodenum (200×, scale bar 50 μm); goblet cells are labeled deep purple. **(e)** Goblet cells/crypt were determined (n = 3, 3 crypts per mouse, data represent the mean ± SD, two-way ANOVA with Tukey’s post-test). **(f)** The relative expression of IL-4 and IL-13 was detected by qPCR at specific time points (0 dpi, 3 dpi, 8 dpi, 17 dpi, 40 dpi). mRNA expression of different groups (normalized to GAPDH) relative to the 0 dpi-no-ABX group (n = 3, data represent the mean ± SD, two-way ANOVA with Tukey’s post-test). All statistical differences are expressed as ns = no significant difference, *p<0.05, **p<0.01, ***p<0.001, ****p<0.0001.

It is well known that mucosal immunity and the corresponding secretion of type II cytokines play an important role in deworming [39]. We first measured the expression of MUC2 in the small intestine of each group. qPCR results showed that the ABX group had lower expression levels of the MUC2 gene than the no-ABX group due to disturbance of the gut microbiota **(Fig 4A).** In addition, PAS and histochemical staining of intestinal sections showed that the ABX group had decreased goblet cell numbers and less mucus secretion **(Fig 4B–4E)**. These results suggest that microbiota-mediated mucus production is one of the key factors affecting excretion. Then, we detected IL-4 and IL-13 levels and found that the ABX group had significantly lower levels than the no-ABX group, showing a trend of reduced Th2 responses (**Fig 4F)**.

### 3.5 Perturbation of the gut microbiota destabilizes the CD4+ T-cell population

**Fig 5:**
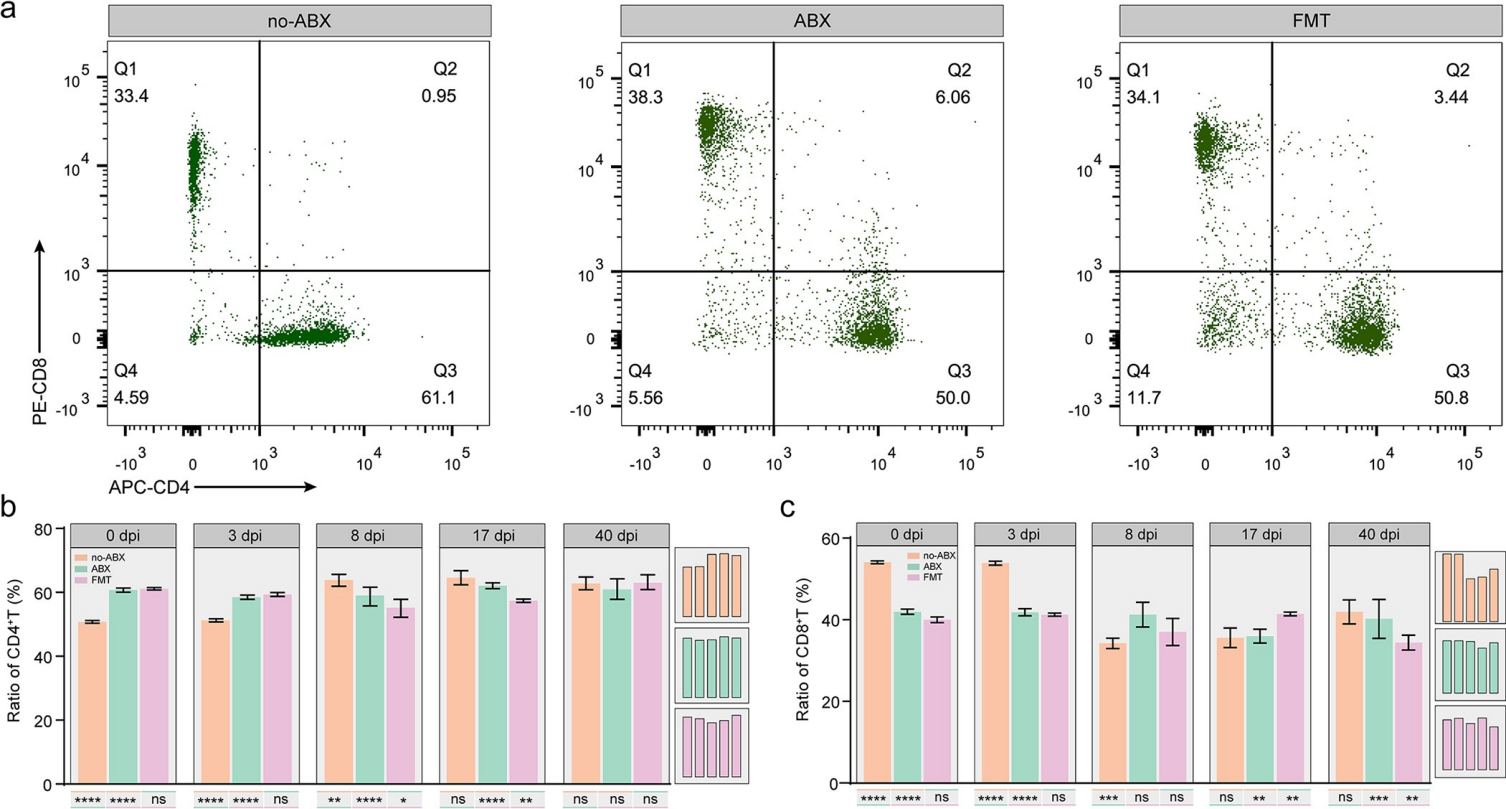
T-cell responses to *T*. *spiralis* infection in the presence or absence of microbiota homeostasis. **(a)** A single cell suspension from MLNs was stained with CD3, CD8 and CD4 antibodies and gated based on CD3+ cells. Representatives from different groups (no-ABX, ABX, FMT) at 8 dpi are shown. The horizontal axis is CD4 cells, and the vertical axis is CD8 cells. **(b-c)** The proportion of CD4+ T cells **(b)** and CD8+ T cells **(c)** in different groups at specific time points (0 dpi, 3 dpi, 8 dpi, 17 dpi, 40 dpi) (n = 3, data represent the mean ± SD, two-way ANOVA with Tukey’s post-test). All statistical differences are expressed as ns = no significant difference, *p<0.05, **p<0.01, ***p<0.001, ****p<0.0001.

To elucidate the effect of the gut microbiota on *T*. *spiralis*-induced T-cell immune responses, we isolated T cells from the mesenteric lymph nodes (MLNs) of the three groups and found that the proportion of CD4+ T cells gradually increased from the third day to a peak on the 17th day, which means that *T*. *spiralis* infection can elicit a T-cell immune response after 3 days **(Fig 5A and 5B)**. It is well known that the immune response mediated by T cells is very important for the expulsion of AD during the intestinal phase, which depends on CD4+ T cells [40]. The proportion of CD4+ T cells increased significantly on the eighth day after infection. The ABX group exhibited fewer CD4+ T cells. CD8+ T cells play a key role in worm expulsion via the subset of CD8+ cytotoxic T cells (Tc), which can activate mononuclear phagocytes to kill parasites [41]. However, this population was strongly inhibited during *T*. *spiralis* infection, which was favorable for parasite survival, and the CD8+ T-cell ratio increased was higher than that in the no-ABX group, but there was no significant difference in the levels in the ABX group at different time points **(Figs 5 and S5)**. Therefore, the current results suggest that the gut microbiota can affect the expulsion of *T*. *spiralis* by affecting the proportion of CD4+ T cells.

### 3.6 Intestinal IgA levels after host infection are dependent on the gut microbiota

**Fig 6:**
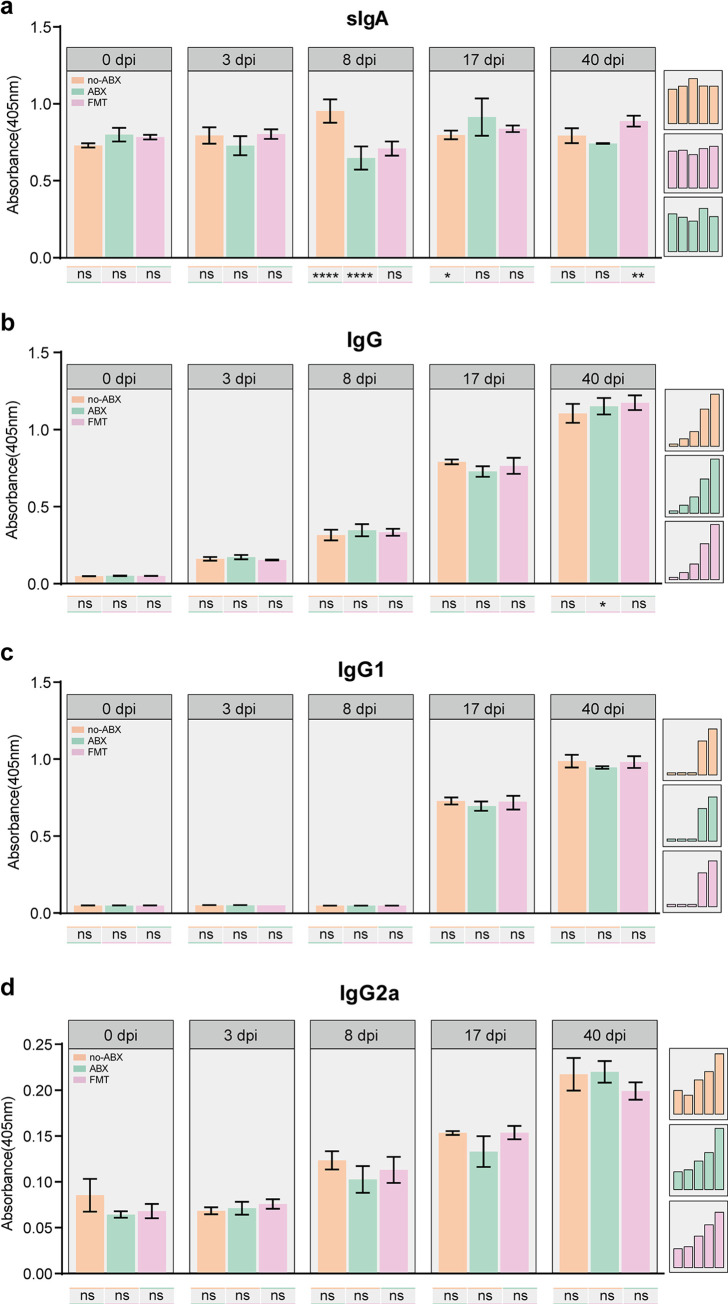
Antibody levels in the host following *T*. *spiralis* infection in the presence or absence of microbiota homeostasis. The OD resulting from the analysis of sIgA **(a)**, IgG **(b)**, IgG2a **(c)**, and IgG1 **(d)** levels in different groups (no-ABX, ABX, FMT) at specific time points (0 dpi, 3 dpi, 8 dpi, 17 dpi, 40 dpi) was measured by ELISA (n = 3, data represent mean ± SD, two-way ANOVA with Tukey’s post-test). All statistical differences are expressed as ns = no significant difference, *p<0.05, **p<0.01, ***p<0.001, ****p<0.0001).

In addition to evaluating of cellular immune responses, we analyzed sIgA and humoral immune responses after infection. We found that the levels of sIgA were significantly increased at 8 dpi and 17 dpi, and the ABX group showed lower levels than the no-ABX group at 8 dpi **(Fig 6A)**. In contrast, the levels of IgG, IgG2a, and IgG1 increased significantly after 8 dpi, but there was no significant difference among the groups **(Fig 6B and 6D)**. These results further suggest that the presence or absence of the gut microbiota has no effect on humoral immunity after *T*. *spiralis* infection but has an effect on gut immunity.

## Discussion

The gut microbiota has been of interest for researchers since its discovery because of its close relationship with the host [42]. Studies have reported that the gut microbiota can regulate various biological activities of the host, and the disruption of homeostasis can cause a variety of diseases [28,43]. Some parasitic worms occupy the gastrointestinal niche during all or part of their life cycle. Therefore, the gut microbiota is in close contact with gastrointestinal worms [44]. Previous studies on the relationship between intestinal parasites and their hosts have focused on parasites directly regulating the functions of host cells, such as immune cells and epithelial cells, through functional proteins released by parasites [13,45,46]. However, this immune-centric view of host-parasite interactions should not ignore the possibility that the microbiome is involved in tripartite relationships. Many studies have reported that after intestinal worm infection, the diversity and community composition of the gut microbiota change [47,48]. It has been reported that after *Trichuris muris* infection, the diversity of the gut microbiota increases significantly, the proportion of Clostridium among the entire gut microbiota decreases, and the proportion of *Bacteroides* increases [16,49,50]. However, when worms such as *Ascaris suum* and *Ascaris lumbricoides* infect the intestine, they increase the proportions of *Firmicutes* and *Lactobacillus*, which play an important role in the digestion of carbohydrates [51]. The metabolism of carbohydrates by these microbiota is a key process for providing nutrients and energy to the host [52]. Although there have been reports of changes in the gut microbiota after infection with *T*. *spiralis*, the roles of the gut microbiota in *T*. *spiralis* infection remain unclear [53]. Based on sequencing analysis and knowledge of the lifecycle of *T*. *spiralis*, such as that 3 dpi is when it develops into AD, the 8 dpi is when the new larvae begin to migrate, 17 dpi is when all the AD in the intestine are expulsed, and 40 dpi is when the ML successfully parasitizes the muscles, this study analyzed the diversity and community changes in the gut microbiota during different stages of infection with *T*. *spiralis* to identify different microbes and explore the possible role of the microbiome in the invasion of the host and colonization of *T*. *spiralis*.

Our study showed that the diversity of microbiota in the Ts3 and Ts8 groups decreased, along with intestinal inflammation caused by *T*. *spiralis* invasion of the small intestine. In addition, the Ts3 and Ts8 groups also exhibited a variety of bacteria with different altered abundance than in the control group. Proteobacteria, which are markers of intestinal disease, showed significantly increased abundance in these groups, which was most likely related to the decrease in the abundance of beneficial bacteria due to the disruption of intestinal homeostasis by *T*. *spiralis* invasion. In addition, Ruminococcus abundance was higher in these two groups. As an important inflammatory marker, Ruminococcus can produce metabolites in the form of mannan polysaccharides, and can also stimulate immune cells to produce TNF-α to cause inflammation [54]. Metabolite analysis of the Ts8 group also showed that Proteobacteria and Ruminococcus levels were positively correlated with the levels of a variety of ceramides. An increased in ceramide levels is known to exacerbate inflammation [55], disrupt the mitochondrial membrane structure, increase membrane permeability and trigger apoptosis [56,57]. Conversely, ceramides levels were also significantly negatively correlated with *Odoribacteraceae* abundance, which has been reported to produce secondary bile acids with good anti-inflammatory and antibacterial activities [38]. The intestinal tract showed severe inflammation during the early stage of *T*. *spiralis* infection, which was also consistent with shortened villi and crypt hyperplasia in intestinal pathological sections. With the colonization of *T*. *spiralis* in the intestine, the abundance and diversity of the microbiome in the Ts8 group recovered, and the proportions of most pathogenic bacteria also decreased slightly, but they did not recover to the baseline level. It seems that *T*. *spirali*s may repair intestinal homeostasis in order to survive. In the Ts17 group, the gut microbiota changed again. There was a clear increase in total bacterial abundance, which we suspect may have been related to immune regulation of *T*. *spiralis*.

Given that the diversity and community structure of the gut microbiota in mice with *T*. *spiralis* infection was different, we are interested in whether the microbiota affects *T*. *spiralis* parasitism and immune regulation of the host. We established a model of the disturbed intestinal microbiota with ABX as is commonly reported to investigate the role of microbiota in the pathogenic infection [28]. Interestingly, ABX treatment caused intestinal inflammation without infection, while FMT treatment did not result in a similar intestinal microenvironment or even a higher cytokine expression than expected in the untreated group. However, inflammation also coincides with the widespread use of antibiotics, which disturbs the gut microbiota and leads to inflammatory bowel disease (IBD) [58]. Although fecal bacteria transplantation is a better treatment for IBD, it is a long-term process [59]. The increase in the levels of inflammatory cytokines during the early period after *T*. *spiralis* infection is also associated with increased permeability of the intestinal barrier due to invasion by *T*. *spiralis*. The destruction of the intestinal barrier caused by worm invasion has been reported to cause bacterial displacement and inflammation [49]. This is also consistent with our research. We found that the expression of TNF-α and IFN-γ in the ABX group was lower than that in the no-ABX group. Infection with *T*. *spiralis* eliminated intestinal inflammation caused by ABX and FMT treatment, which fully demonstrates the immunomodulatory capacity of *T*. *spiralis*. However, at 17 dpi, there was more intense inflammation in the ABX group than in the no-ABX group. We believe that this difference results by the fact that the gut microbiota and its metabolites in the no-ABX group are beneficial to recovery from inflammation as *T*. *spiralis* leaves the intestine, but the ABX group still exhibits an inflammatory state due to disturbance of the microbiota. After entering the ML phase, the microbiome was significantly changed and was even better than that in the CK40 group, which may have been due to remodeling of the microbiome by the parasites after intestinal clearance. Our results showed that the abundance of *Lactobacillaceae* in the Ts40 group was ten-fold higher than that in the other groups. This is consistent with other research reporting that *Lactobacillaceae* can have anti-inflammatory properties through the production of short-chain fatty acids [60]. We suspect that the remission of autoimmune diseases during the later stage of *T*. *spiralis* infection is closely related to this mechanism [61,62]. Of course, this specific mechanism and experimental studies will be the direction of our future research.

Several studies have also reported that the gut microbiota is involved in the growth, development and expulsion of parasites [20,22]. However, there are also some reports to the contrary, indicating that the expulsion of *Hymenolepis diminuta* in mice is independent of the composition of the gut microbiota [63]. Similarly, we report the expulsion of *T*. *spiralis* and investigate its immune-related mechanisms. Treatment of mice with broad-spectrum antibiotics affects *T*. *spiralis* expulsion kinetics, mobilization of local effector cells (e.g., goblet cells), and the development of systemic Th2 responses. Goblet cells are important due to their involvement in helminth expulsion, and it has been reported that impaired expulsion of *Nippostrongylus brasiliensis* in Pou2f3^-/-^ mice can be attributed to reduced goblet cell proliferation [64]. We show that ABX mice have significantly lower ratios of IL-4 and IL-13 in the small intestine than no-ABX mice, as well as reduced goblet cell numbers and mucus production, implying a weakening of mucosal immunity and Th2 responses, which is largely related to the reduction of worms. This is also consistent with recent reports that the gut microbiota plays a major role in driving mucus changes and is associated with helminth burden [39,65]. This phenomenon was related to the decrease in the proportion of CD4+ T cells in the ABX group. The present study showed that the proportion of CD8+ T cells increased slightly after infection and returned to basal levels later in infection in the ABX group. CD4+ T cells are generated during the intestinal phase of *T*. *spiralis* infection after 2–4 dpi and play a key role in expulsion [66,67]. In our study, the highest proportion of CD4+ T cells was seen at 8 dpi, which indicates strong deworming power. Therefore, the ABX group had a more inhibited response, which may have affected the production of cytokines, thereby inhibiting the anthelmintic effect.

In addition to the role of cellular immunity in the expulsion of *T*. *spiralis*, humoral immunity also helps to resist this parasite [68,69]. It was previously reported that the establishment of infectious *T*. *spiralis* in the mouse gut can be prevented when a sufficient mucosal IgA response is induced [70]. Our results showed that the levels of IgG, IgG2a, and IgG1 after infection increased significantly over time, but there was no difference between the treatment groups, indicating that the microbiota did not significantly affect the changes in humoral immunity caused by *T*. *spiralis*. Conversely, the levels of sIgA were significantly lower in the ABX group at 8 dpi than in the no-ABX group, which may have been due to ineffective mucosal immunity, and this result was also consistent with reduced goblet cell counts and mucus secretion. Overall, we have determined that the abundance of bacteria that produce a large number of inflammatory metabolites is increased during the early stage of *T*. *spiralis* infection. During the later stage of infection, the proportion of *Lactobacillus* is increased, which endows *T*. *spiralis* with an anti-inflammatory effect. ABX inhibited the expulsion of *T*. *spiralis* and led to an increase in the parasite load. The participation of the microbiota in the parasitism of *T*. *spiralis* in the host may be mediated by the Th2 response and IL-4. The presence or absence of the gut microbiota had no effect on humoral immunity after *T*. *spiralis* infection but influenced gut immunity. Due to the direct contact between *T*. *spiralis* and gut microbiota during the early stage of infection, many studies focused on the interaction of among *T*. *spiralis*, the microbiota and the host during the intestinal stage. However, during the ML stage, in addition to studying the interaction between *T*. *spiralis* and the host mediated by the excretion and secretion of products, whether the gut microbiota affects the formation of cyst needs further investigation. At the same time, there are some limitations in this study. Although the role of the gut microbiota in expulsion was identified in this study, the community of the gut microbiota after infection was not measured, as a result, the effectiveness of colonization and the important role that certain bacteria play are unknown.Sequencing of the FMT supernatant microbiota will contribute to further interpretation and is expected to be carried out in subsequent studies.

Many layers of crosstalk between parasites, the gut microbiota and the host’s immune system form a complex ecosystem, changes in one of the components will provoke responses by the remaining components. It is hard to determine whether these interactions are due to immune regulation by helminth-to-microbiota effects, immune regulation by microbiota-to-helminth effects, or both. Meantime, host genetics and environmental factors are related to the intestinal microenvironment and also affect the intensity of helminth infection. Thus, the multidirectional cross-talk of these associations requires further investigation as an integrated system. The identification of the specific gut microbiota and/or their metabolites involved in helminth-microbiota interactions will facilitate the diagnosis of parasitic infections caused by helminth, and assist in the development of new strategies for controlling parasites infections and inflammatory diseases. In addition, it is necessary to investigate the interaction between intestinal parasites and the microbiota in its natural state, which will help to translate laboratory findings into clinical application.

## Conclusion

In conclusion, this study examined the gut microbiota during various stages of *T*. *spiralis* infection by 16S rRNA sequencing, and showed that Proteobacteria, Ruminococcus, etc., are likely to participate in the intestinal inflammatory response by producing ceramides after infection with *T*. *spiralis*. The gut microbiota can expel parasites by participating in mucosal immunity and Th2 immune responses. CD4+ T cells, IL-4, and sIgA are the major molecules. These findings also further explain the relationship among parasites, the gut microbiota, and the host and makes significant progress in the understanding of host-parasite interactions.

## Supporting information

S1 Fig **(a)** Flower plot of OTUs in each sample. The core of the petal shows the number of common OTUs of each sample, and the end of the petal shows the unique OTUs of each sample. **(b)** Rarefaction curves demonstrate the increase in the number of OTUs detected as the number of samples sequenced increases. **(c)** Differences in the relative abundance of the top 15 families in different groups. All statistical differences are expressed as* p<0.05, ** p<0.01.
(TIF)

S2 Fig Experimental treatment schedule of mice. Male C57BL/6 mice were treated as shown in the panel (no-ABX, no pretreatment was performed; ABX, cocktail antibiotic treatment; FMT, fecal bacteria transplantation was performed after treatment with a cocktail of antibiotics; ML, muscle larvae of *T*. *spiralis*). The mice in this figure was modified from Servier Medical Art (http://smart.servier.com/), licensed under a Creative Common Attribution 3.0 Generic License (https://creativecommons.org/licenses/by/3.0/).
(TIF)

S3 Fig The distribution of the Shannon index in CK and ABX and the *p* value of the difference in the diversity index between groups. (TIF)

S4 Fig The ability of a single female to produce newborn larvae. A single female AD at 6 dpi produces the number of newborn larvae. (n = 8, data represent the mean ± SD, T-test). The statistical differences are expressed as ns = no significant difference.
(TIF)

S5 Fig T lymphocyte gating strategy and scatter plots of different treatment groups at 0 dpi, 3 dpi, 17 dpi, and 40 dpi. (TIF)

S1 Table First round PCR system for 16S amplification. (DOCX)

S2 Table First round PCR program for 16S amplification. (DOCX)

S3 Table Second round PCR system for 16S amplification. (DOCX)

S4 Table Second round PCR program for 16S amplification. (DOCX)

S5 Table Different lipid metabolites between the control group and *Trichinella spiralis* group. (DOCX)

S1 Code The analysis and plotting code in Fig 1. (DOCX)
