# Supplementary figures and images for: The gut microbiota contributes to changes in the host immune response induced by Trichinella spiralis
Source: PLoS Negl Trop Dis. 2023 Aug 16;17(8):e0011479. doi: 10.1371/journal.pntd.0011479 (PMC10431649; doi:10.1371/journal.pntd.0011479)

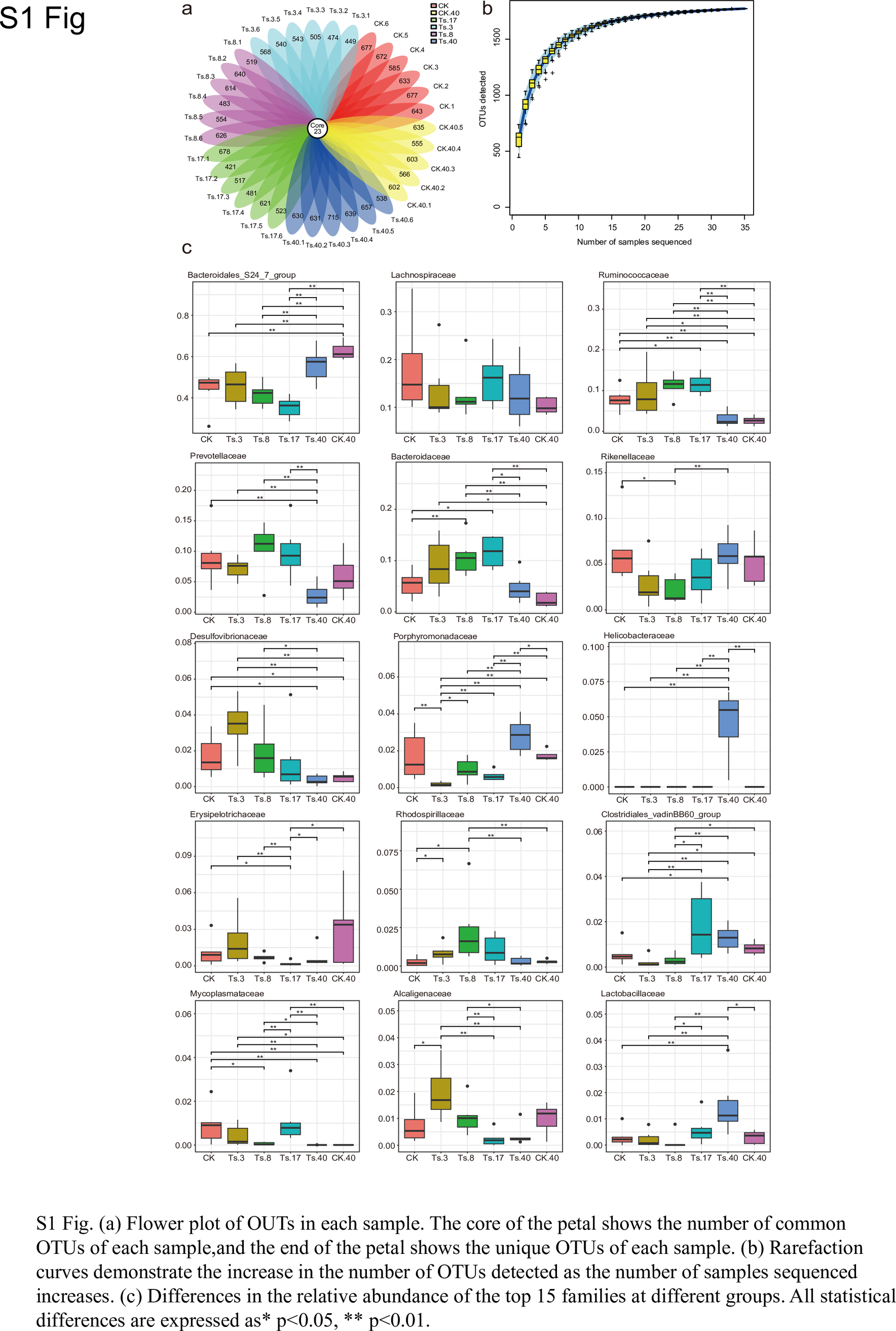

Supplement: S1 Fig — (a) Flower plot of OTUs in each sample. The core of the petal shows the number of common OTUs of each sample, and the end of the petal shows the unique OTUs of each sample. (b) Rarefaction curves demonstrate the increase in the number of OTUs detected as the number of samples sequenced increases. (c) Differences in the relative abundance of the top 15 families in different groups. All statistical differences are expressed as* p<0.05, ** p<0.01. (TIF) [file pntd.0011479.s001.tif]

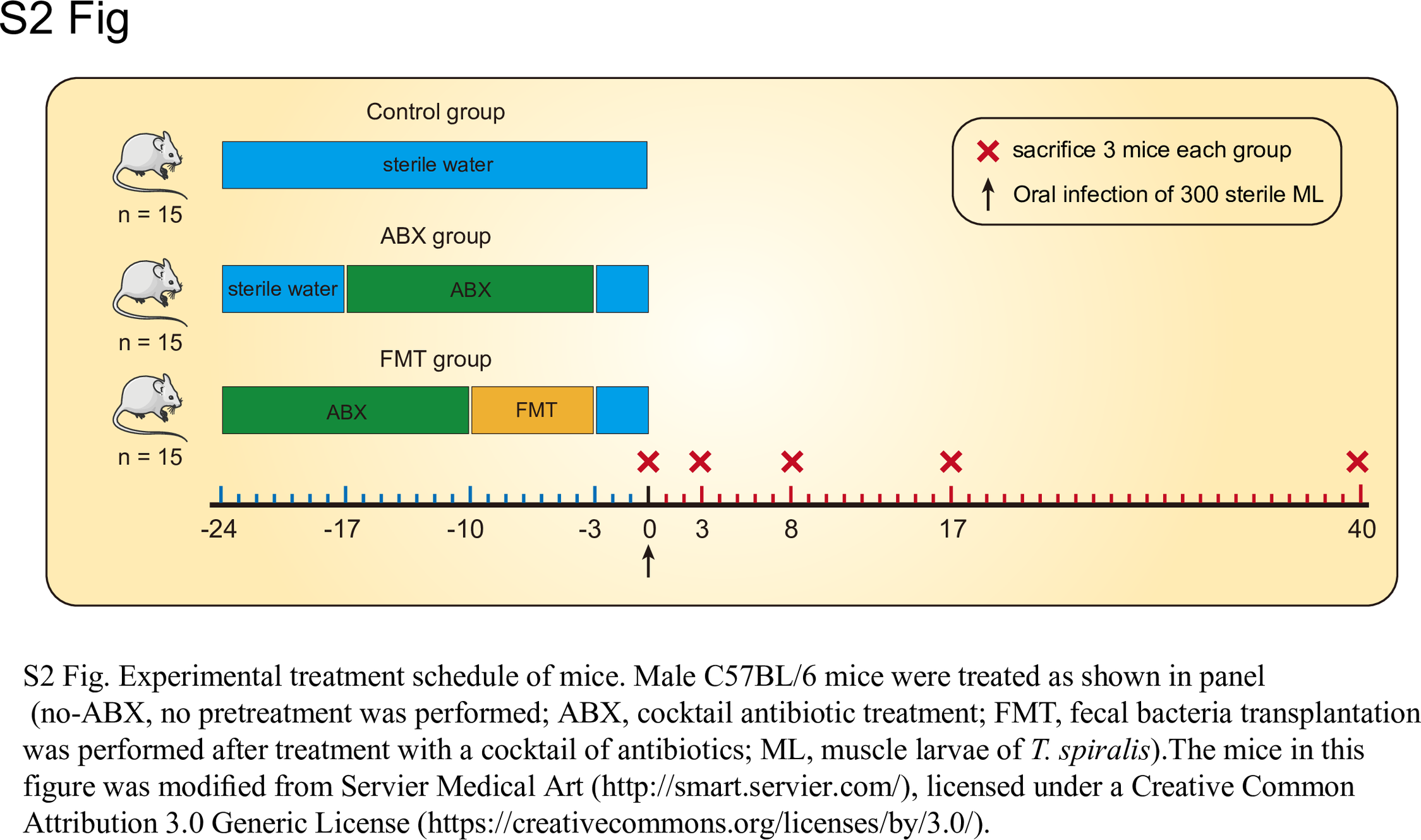

Supplement: S2 Fig — Male C57BL/6 mice were treated as shown in the panel (no-ABX, no pretreatment was performed; ABX, cocktail antibiotic treatment; FMT, fecal bacteria transplantation was performed after treatment with a cocktail of antibiotics; ML, muscle larvae of T. spiralis). The mice in this figure was modified from Servier Medical Art (http://smart.servier.com/), licensed under a Creative Common Attribution 3.0 Generic License (https://creativecommons.org/licenses/by/3.0/). (TIF) [file pntd.0011479.s002.tif]

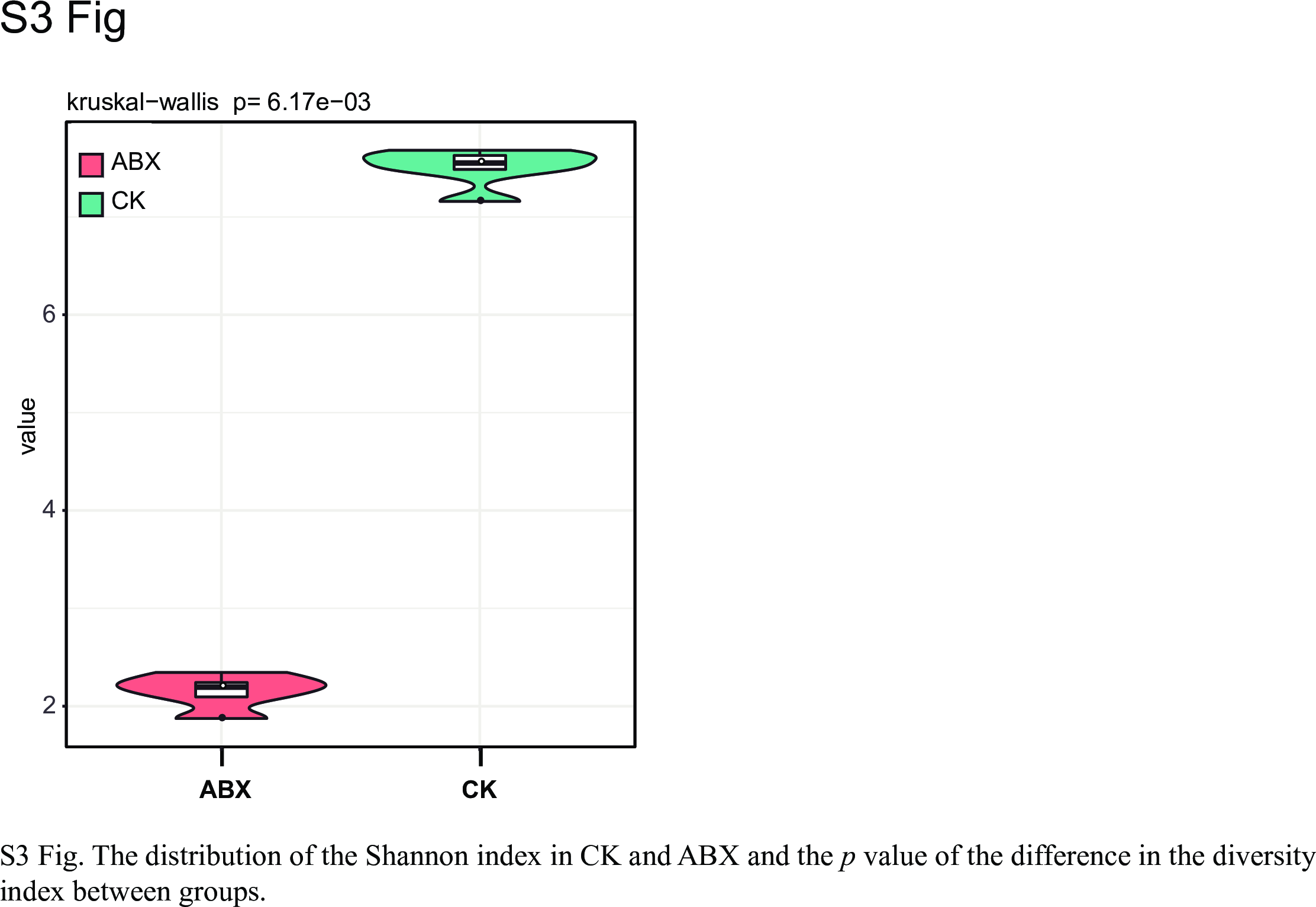

Supplement: S3 Fig — (TIF) [file pntd.0011479.s003.tif]

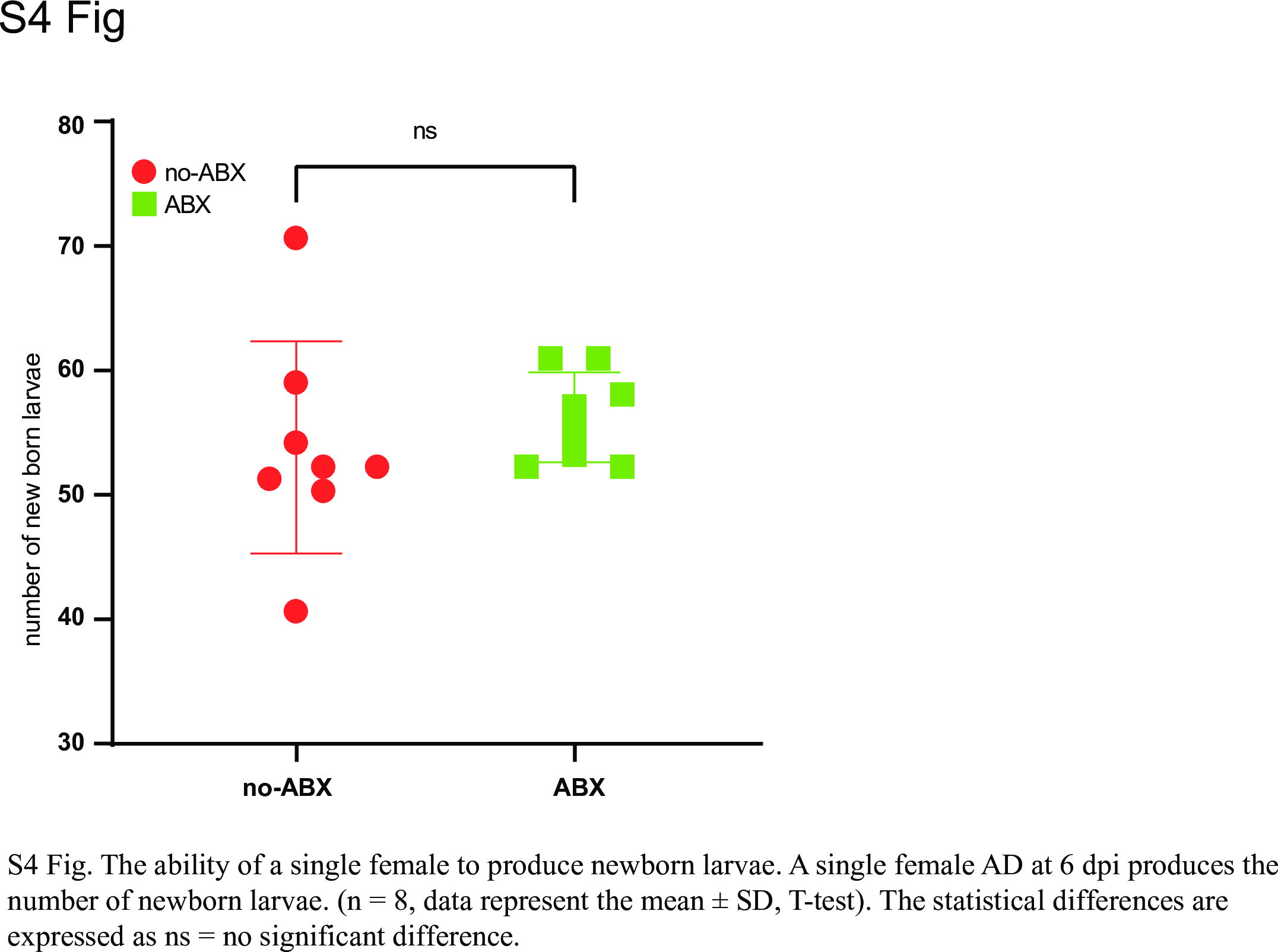

Supplement: S4 Fig — A single female AD at 6 dpi produces the number of newborn larvae. (n = 8, data represent the mean ± SD, T-test). The statistical differences are expressed as ns = no significant difference. (TIF) [file pntd.0011479.s004.tif]

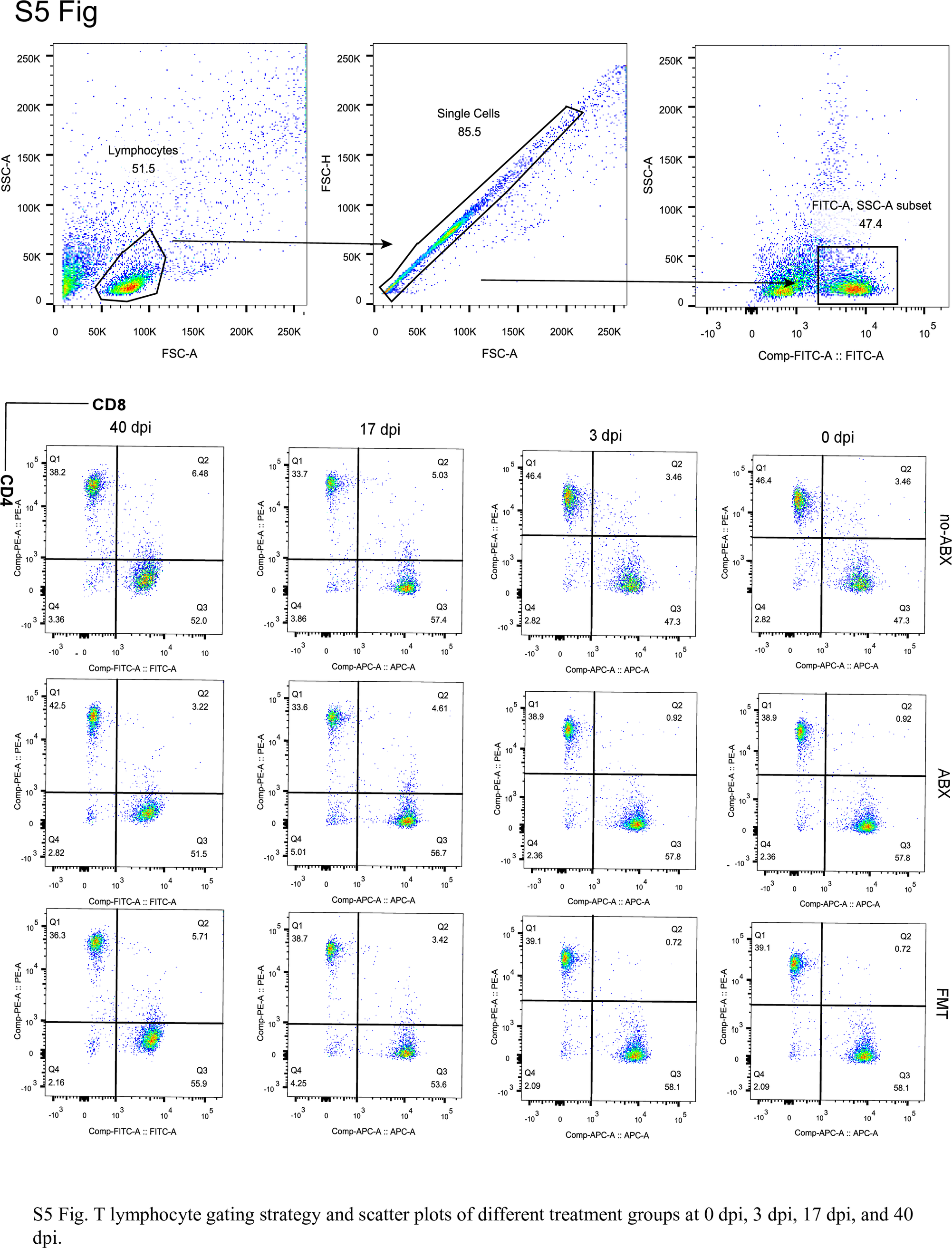

Supplement: S5 Fig — (TIF) [file pntd.0011479.s005.tif]
