# Supplementary material for: The gut microbiota contributes to changes in the host immune response induced by Trichinella spiralis
Source: PLoS Negl Trop Dis. 2023 Aug 16;17(8):e0011479. doi: 10.1371/journal.pntd.0011479 (PMC10431649; doi:10.1371/journal.pntd.0011479)
Supplement: S2 Table — (DOCX) [file pntd.0011479.s007.docx]

**S2 Table: First round PCR program for 16S amplification.**

| **Temperature** | **Time** | **cycle(s)** |
| --- | --- | --- |
| 94 ℃ | 5 min | 1 |
| 94 ℃ | 30 s | 26 |
| 56 ℃ | 30 s |  |
| 72 ℃ | 20 s |  |
| 72℃ | 5 min | 1 |
| 4 ℃ | hold | - |
