# Supplementary material for: The gut microbiota contributes to changes in the host immune response induced by Trichinella spiralis
Source: PLoS Negl Trop Dis. 2023 Aug 16;17(8):e0011479. doi: 10.1371/journal.pntd.0011479 (PMC10431649; doi:10.1371/journal.pntd.0011479)
Supplement: S3 Table — (DOCX) [file pntd.0011479.s008.docx]

**S3 Table: Second round PCR system for 16S amplification.**

| **Reagent** | **volume (μL)** |
| --- | --- |
| 2×Gflex PCR Buffer | 15 |
| Adapter i5 | 1 |
| Adapter i7 | 1 |
| First-round product | ≥1(50 ng) |
| Tks Gflex DNA Polymerase (1.25U/μL) | 0.6 |
| H_2_O | up to 30 |
