# Supplementary material for: The gut microbiota contributes to changes in the host immune response induced by Trichinella spiralis
Source: PLoS Negl Trop Dis. 2023 Aug 16;17(8):e0011479. doi: 10.1371/journal.pntd.0011479 (PMC10431649; doi:10.1371/journal.pntd.0011479)
Supplement: S5 Table — (DOCX) [file pntd.0011479.s010.docx]

**S5 Table: Different lipid metabolites between control group and *Trichinella spiralis* group.**

| **Lipid** | **Class** | **adj.P-value** | **T.spiralis6** | **T.spiralis5** | **T.spiralis4** | **T.spiralis3** | **T.spiralis2** | **T.spiralis1** | **control6** | **control5** | **control4** | **control3** | **control2** | **control1** |
| --- | --- | --- | --- | --- | --- | --- | --- | --- | --- | --- | --- | --- | --- | --- |
| FA(22:4) | FA | 0.00691 | 365.738 | 359.625 | 648.683 | 126.433 | 369.807 | 209.16 | 875.817 | 964.911 | 570.091 | 980.026 | 775.538 | 817.89 |
| LPE(15:0) | LPE | 0.11071 | 1005.22 | 315.209 | 441.421 | 706.651 | 1017.29 | 418.32 | 293.075 | 276.278 | 426.694 | 274.827 | 279.367 | 461.867 |
| SM(d34:1) | SM | 0.00625 | 160.162 | 384.311 | 216.612 | 212.423 | 362.559 | 240.053 | 52.7654 | 92.7294 | 57.689 | 44.5317 | 49.1818 | 87.9298 |
| SM(d18:1/16:0)+CH3COO | SM | 0.0019 | 116.453 | 266.495 | 180.366 | 194.806 | 230.967 | 199.864 | 9.0223 | 20.0638 | 33.7508 | 47.9523 | 47.2819 | 20.0142 |
| MG(16:2) | MG | 0.00532 | 189.115 | 122.907 | 187.34 | 120.805 | 52.6198 | 166.488 | 6.65627 | 12.5492 | 20.5624 | 19.4075 | 4.60492 | 45.5059 |
| DG(34:2p) | DG | 0.00779 | 121.502 | 61.091 | 138.017 | 66.4836 | 219.187 | 158.745 | 8.22245 | 6.0552 | 14.2366 | 1.33276 | 3.88442 | 6.6174 |
| Cer(d18:1/16:0) | Cer | 0.0025 | 153.7 | 160.47 | 88.6007 | 103.515 | 137.542 | 111.862 | 21.6398 | 56.905 | 41.4453 | 17.7839 | 12.4987 | 34.8325 |
| DG(34:3p) | DG | 0.00641 | 149.953 | 38.4311 | 128.797 | 54.1598 | 120.102 | 132.933 | 3.91545 | 5.11913 | 16.707 | 1.77784 | 3.94707 | 4.35117 |
| TG(14:0e/15:0/17:1) | TG | 0.07586 | 127.862 | 54.3837 | 49.4696 | 38.9172 | 6.81581 | 88.1485 | 153.262 | 244.841 | 214.192 | 94.2416 | 53.5674 | 277.387 |
| OAHFA(20:1/34:1) | OAHFA | 0.02711 | 7.97646 | 18.1962 | 27.2131 | 15.6196 | 10.2422 | 7.44635 | 33.2265 | 99.1714 | 41.795 | 139.062 | 184.515 | 121.625 |
| LPE(18:0p) | LPE | 0.04417 | 76.0646 | 176.231 | 49.8379 | 94.5384 | 159.601 | 206.062 | 31.1818 | 37.9367 | 16.5336 | 41.6586 | 35.7497 | 74.2836 |
| DG(34:1p) | DG | 0.01404 | 127.025 | 49.3079 | 44.2006 | 30.6473 | 84.8223 | 84.5348 | 3.97139 | 4.94362 | 14.9934 | 0.98517 | 1.44522 | 4.42369 |
| OAHFA(18:1/18:1) | OAHFA | 0.02729 | 142.256 | 78.3725 | 183.53 | 90.1457 | 117.949 | 69.1003 | 32.8857 | 36.0811 | 65.4031 | 32.6675 | 57.9491 | 41.568 |
| So(d16:1) | So | 0.04807 | 79.8299 | 111.487 | 97.1829 | 86.2665 | 46.1643 | 72.7903 | 124.362 | 186.921 | 87.3903 | 157.207 | 233.379 | 140.506 |
| LPG(18:1p) | LPG | 0.06322 | 112.638 | 119.923 | 210.427 | 162.912 | 96.1498 | 60.7339 | 49.4138 | 53.4 | 102.477 | 27.6324 | 53.3363 | 51.96 |
| OAHFA(22:1/34:1) | OAHFA | 0.02112 | 4.13195 | 9.15595 | 11.0822 | 7.9516 | 4.66644 | 4.0765 | 21.1286 | 62.8842 | 26.0563 | 83.0175 | 109.267 | 71.7818 |
| SM(d42:2) | SM | 0.00263 | 36.8188 | 67.617 | 54.2995 | 47.5114 | 73.8629 | 40.9123 | 6.50711 | 19.5405 | 8.93894 | 9.90054 | 10.2436 | 6.41797 |
| TG(16:0e/15:0/16:1) | TG | 0.10747 | 83.1771 | 37.1622 | 31.1746 | 28.215 | 4.77407 | 52.5277 | 99.005 | 132.22 | 130.8 | 49.917 | 26.3765 | 188.551 |
| OAHFA(18:2/34:1) | OAHFA | 0.10747 | 12.8906 | 25.36 | 40.9778 | 26.5472 | 29.5845 | 12.5998 | 30.6706 | 51.7506 | 37.2483 | 105.495 | 200.948 | 116.044 |
| PE(16:0/18:1) | PE | 0.07238 | 25.8036 | 15.4739 | 28.1624 | 30.3669 | 20.6313 | 7.96657 | 129.157 | 102.264 | 121.363 | 38.9613 | 20.6565 | 47.7263 |
| OAHFA(18:1/34:1) | OAHFA | 0.06271 | 16.9916 | 31.0911 | 54.7425 | 27.5021 | 37.2402 | 13.0773 | 36.9751 | 76.4919 | 44.7679 | 105.795 | 155.684 | 101.996 |
| So(d16:0) | So | 0.09781 | 67.278 | 90.2769 | 64.3983 | 107.022 | 37.8322 | 68.7894 | 104.226 | 166.737 | 73.682 | 110.604 | 211.45 | 113.856 |
| DG(36:4p) | DG | 0.00641 | 32.3002 | 38.0686 | 25.1739 | 21.5666 | 70.1848 | 48.6559 | 1.51868 | 1.7719 | 2.55602 | 0.35155 | 0.85084 | 1.64417 |
| Cer(d18:0/22:0) | Cer | 0.0069 | 42.6764 | 79.2189 | 58.3975 | 39.5658 | 54.4214 | 42.7191 | 7.88684 | 22.2317 | 7.18257 | 11.4333 | 24.1837 | 27.5574 |
| OAHFA(21:1/34:1) | OAHFA | 0.03676 | 1.78345 | 7.41917 | 7.67041 | 5.00798 | 2.90895 | 2.79883 | 14.6905 | 30.5143 | 17.8372 | 75.2252 | 85.338 | 63.8916 |
| Cer(d18:0/16:0) | Cer | 0.02382 | 83.4691 | 18.0529 | 22.4666 | 52.1394 | 63.7106 | 57.9451 | 7.20859 | 11.3212 | 14.5331 | 7.16518 | 5.2383 | 12.8331 |
| DG(32:1p) | DG | 0.00494 | 42.0069 | 20.3032 | 31.6137 | 21.2423 | 52.2445 | 54.3346 | 2.49843 | 2.58374 | 6.21156 | 0.70125 | 0.85481 | 2.90078 |
| OAHFA(20:1/33:1) | OAHFA | 0.04856 | 3.74222 | 11.2764 | 13.6082 | 9.34046 | 5.94046 | 3.68944 | 15.5655 | 30.9266 | 19.0613 | 73.7267 | 89.086 | 63.1218 |
| PG(48:2) | PG | 0.03676 | 0.25522 | 2.3761 | 9.35077 | 2.74119 | 0.27547 | 0.49238 | 73.4391 | 55.8741 | 50.8885 | 15.2781 | 15.4437 | 18.4634 |
| Cer(d18:1/16:0+O) | Cer | 0.00666 | 61.4205 | 60.3659 | 58.3975 | 43.1332 | 70.6352 | 31.6199 | 24.6114 | 29.2522 | 18.5633 | 18.9933 | 25.8439 | 22.481 |
| TG(14:0e/15:0/16:1) | TG | 0.08145 | 34.6432 | 13.5415 | 11.3868 | 10.4428 | 2.08678 | 22.0694 | 52.7654 | 70.4978 | 76.3951 | 21.7481 | 13.1256 | 103.703 |
| PE(15:0/14:0) | PE | 0.04639 | 55.7303 | 12.6351 | 28.3938 | 12.1292 | 18.2405 | 15.1001 | 62.2743 | 86.5865 | 78.6798 | 55.9236 | 27.0343 | 48.588 |
| Cer(d36:4) | Cer | 0.09853 | 51.0443 | 78.675 | 42.0052 | 69.078 | 62.3781 | 139.386 | 15.0652 | 37.4428 | 16.9926 | 29.6188 | 37.2779 | 64.5423 |
| Cer(d20:1/20:0) | Cer | 0.00532 | 34.4758 | 56.7403 | 39.3708 | 35.3498 | 37.2317 | 27.7481 | 9.06148 | 23.4603 | 7.33964 | 8.55423 | 10.7135 | 16.2806 |
| SM(d40:1) | SM | 0.00286 | 21.9239 | 44.232 | 30.1501 | 28.5393 | 42.1859 | 26.0703 | 4.04597 | 13.0172 | 4.46947 | 6.87653 | 7.39293 | 6.68992 |
| Cer(d42:0+O) | Cer | 0.0019 | 52.5048 | 44.2726 | 52.2111 | 39.5342 | 53.33 | 42.9166 | 15.964 | 26.3907 | 16.8199 | 25.9191 | 21.4631 | 29.8289 |
| PE(15:0/18:2) | PE | 0.03852 | 29.8425 | 8.77827 | 19.9351 | 9.5002 | 20.8908 | 10.2391 | 53.5032 | 67.4201 | 51.2383 | 29.434 | 16.1221 | 64.8538 |
| SM(d42:2)+CH3COO | SM | 0.0019 | 22.8867 | 24.7869 | 26.8966 | 26.1652 | 38.927 | 21.2259 | 2.43739 | 5.73028 | 4.3498 | 6.56492 | 3.97712 | 2.68635 |
| DG(36:5p) | DG | 0.01582 | 31.1286 | 15.4631 | 18.5877 | 12.0805 | 45.0384 | 47.3653 | 1.11991 | 2.18396 | 1.34875 | 0.41053 | 0.70133 | 1.86738 |
| Cer(d40:1+O) | Cer | 0.0019 | 37.0226 | 31.3777 | 42.56 | 29.221 | 42.69 | 26.8035 | 13.6696 | 11.5187 | 15.1837 | 11.91 | 8.46159 | 14.6774 |
| Cer(d32:0) | Cer | 0.06101 | 44.6847 | 58.9156 | 39.2244 | 51.5653 | 31.4518 | 42.0738 | 62.2743 | 92.1444 | 45.2659 | 67.5225 | 104.629 | 70.1626 |
| PEt(19:0) | PEt | 0.0659 | 43.0808 | 36.9655 | 89.3917 | 57.296 | 38.2783 | 23.0851 | 19.0839 | 15.7459 | 33.7508 | 10.2278 | 17.8075 | 15.1068 |
| Cer(d20:0/20:0) | Cer | 0.00286 | 26.028 | 24.6436 | 28.4788 | 19.091 | 37.6295 | 21.2259 | 2.31838 | 5.58233 | 3.24915 | 7.63112 | 9.7404 | 10.4899 |
| PG(50:3) | PG | 0.04773 | 0.24308 | 1.61853 | 3.21 | 2.54388 | 0.93378 | 1.2E-05 | 55.3775 | 33.1946 | 30.4282 | 7.48535 | 13.1346 | 11.9388 |
| LPE(14:0) | LPE | 0.12894 | 87.7323 | 34.3865 | 62.8115 | 73.9119 | 97.7069 | 40.2827 | 47.5395 | 40.8232 | 67.3268 | 12.851 | 27.4632 | 19.4369 |
| DG(36:3p) | DG | 0.0177 | 19.5809 | 43.1444 | 18.1486 | 15.1291 | 14.6375 | 10.9702 | 0.11069 | 0.18807 | 2.05624 | 0.07023 | 0.05388 | 0.30081 |
| DG(30:1e) | DG | 0.11535 | 38.4924 | 14.8649 | 25.1739 | 11.5779 | 6.05766 | 13.8095 | 49.7822 | 64.9398 | 67.9702 | 17.357 | 10.087 | 64.9049 |
| DG(32:0p) | DG | 0.02146 | 30.1245 | 17.5478 | 16.3923 | 9.24284 | 43.2368 | 12.648 | 0.64639 | 0.5828 | 2.19904 | 0.15673 | 0.28309 | 0.51864 |
| Cer(d17:0/16:0+O) | Cer | 0.11535 | 38.1577 | 14.8649 | 25.3202 | 11.5779 | 6.05015 | 13.8095 | 49.9686 | 64.9398 | 67.9702 | 17.212 | 10.087 | 64.1797 |
| PG(49:2) | PG | 0.11779 | 1.0955 | 5.51702 | 19.6187 | 13.3972 | 2.83034 | 1.01908 | 65.601 | 54.0185 | 46.3418 | 9.78497 | 4.07104 | 18.1923 |
| SM(d36:1) | SM | 0.01211 | 10.0917 | 26.648 | 21.2222 | 13.3778 | 35.8055 | 14.0676 | 2.16282 | 1.14775 | 1.79921 | 2.09196 | 0.52607 | 3.26337 |
| OAHFA(22:3/18:0) | OAHFA | 0.0099 | 41.7346 | 31.8075 | 22.6248 | 42.2081 | 24.7835 | 33.9304 | 6.09035 | 17.0208 | 14.3863 | 18.0605 | 13.5345 | 18.0281 |
| OAHFA(18:0/34:1) | OAHFA | 0.01611 | 7.27734 | 10.8063 | 14.6699 | 10.8865 | 11.1207 | 4.39841 | 15.476 | 27.2154 | 18.0121 | 37.4628 | 36.6146 | 38.2965 |
| OAHFA(16:0/34:1) | OAHFA | 0.05042 | 9.53298 | 13.4749 | 22.783 | 14.2782 | 19.8528 | 7.89858 | 16.5072 | 29.2772 | 21.5096 | 48.5517 | 51.8948 | 48.6885 |
| OAHFA(18:2/36:1) | OAHFA | 0.04787 | 4.52581 | 9.28366 | 12.5316 | 8.33888 | 7.35304 | 3.88988 | 10.3199 | 24.5351 | 15.2154 | 36.8634 | 53.9129 | 32.1383 |
| PC(15:1/16:0) | PC | 0.09835 | 6.52697 | 6.59855 | 8.78158 | 8.67529 | 4.8116 | 2.41344 | 50.528 | 56.7492 | 33.2711 | 9.25846 | 7.17365 | 14.9209 |
| OAHFA(22:1/35:1) | OAHFA | 0.02733 | 1.06735 | 3.62717 | 3.62665 | 3.11422 | 1.09037 | 1.02752 | 6.76561 | 14.2934 | 9.02265 | 33.2669 | 33.4433 | 28.6742 |
| DG(36:4) | DG | 0.06251 | 24.4343 | 27.5544 | 31.321 | 41.9981 | 1.54632 | 3.65242 | 1.6355 | 2.50622 | 2.39895 | 4.41175 | 0.92953 | 0.80529 |
| OAHFA(18:1/33:1) | OAHFA | 0.06966 | 4.01452 | 9.65883 | 14.2402 | 9.30193 | 9.91867 | 3.72335 | 9.9968 | 17.8423 | 14.6533 | 44.3559 | 49.8766 | 38.104 |
| CerG1(d22:0/18:0+O) | CerG1 | 0.0177 | 17.2379 | 36.2558 | 13.2748 | 25.2962 | 23.7953 | 22.8438 | 5.76131 | 14.0118 | 4.54087 | 8.57495 | 8.86525 | 9.62695 |
| OAHFA(20:0/34:1) | OAHFA | 0.01392 | 2.16727 | 4.37814 | 4.70844 | 3.87986 | 2.9139 | 1.78659 | 7.83818 | 17.6514 | 9.36109 | 28.0315 | 21.9136 | 22.7085 |
| OAHFA(47:2) | OAHFA | 0.07586 | 16.1784 | 17.6231 | 46.99 | 18.979 | 38.6675 | 9.62683 | 4.81607 | 4.66899 | 7.57572 | 8.20374 | 7.21119 | 12.2947 |
| dMePE(16:1/14:0) | dMePE | 0.09781 | 12.0759 | 3.2136 | 9.04271 | 3.8054 | 3.61915 | 1.59568 | 25.7292 | 42.4726 | 41.795 | 15.7041 | 1.50089 | 12.1749 |
| PG(14:0p/16:0) | PG | 0.08582 | 5.60851 | 5.60667 | 12.5108 | 6.03076 | 5.08528 | 1.97246 | 36.8047 | 36.6996 | 32.5267 | 5.84875 | 5.83959 | 18.9885 |
| Cer(d18:0) | Cer | 0.04497 | 13.9744 | 14.6473 | 22.2467 | 23.5125 | 16.0637 | 18.4557 | 30.0185 | 36.5652 | 24.9891 | 50.3312 | 34.7718 | 21.0306 |
| PG(47:1) | PG | 0.03143 | 0.32157 | 0.81787 | 2.72829 | 2.42653 | 0.43104 | 0.43435 | 17.8912 | 26.5969 | 25.0071 | 6.85539 | 2.87151 | 12.6609 |
| PE(16:0/18:2) | PE | 0.07586 | 5.85754 | 3.55307 | 2.15149 | 4.47548 | 3.0476 | 2.1295 | 30.9507 | 39.4904 | 18.1349 | 5.01241 | 11.0267 | 9.88077 |
| DG(16:1/18:3) | DG | 0.01404 | 18.5768 | 12.3088 | 26.6375 | 24.8097 | 8.93261 | 14.0676 | 1.90179 | 1.78865 | 5.08349 | 4.80528 | 2.25998 | 8.08592 |
| DG(34:0p) | DG | 0.0257 | 16.7024 | 12.7258 | 9.35239 | 5.99974 | 29.35 | 7.34356 | 0.2209 | 0.23743 | 0.90727 | 0.02513 | 1.2E-05 | 0.2591 |
| MG(34:1) | MG | 0.02565 | 16.6856 | 12.7258 | 9.32312 | 5.99974 | 29.2749 | 7.34356 | 0.22082 | 0.23264 | 0.90712 | 0.02513 | 1.2E-05 | 0.2591 |
| OAHFA(18:2/35:1) | OAHFA | 0.07799 | 2.56579 | 6.54086 | 8.96339 | 6.30614 | 4.29541 | 2.97782 | 7.94437 | 9.54261 | 9.88378 | 32.0681 | 40.6509 | 31.3685 |
| SM(d40:1)+CH3COO | SM | 0.0019 | 10.7287 | 12.4875 | 12.8812 | 13.0711 | 20.8908 | 11.6685 | 1.31321 | 2.91769 | 2.05349 | 3.37969 | 2.52098 | 1.83085 |
| OAHFA(18:0/33:1) | OAHFA | 0.02645 | 2.93272 | 5.54812 | 7.50267 | 5.98858 | 4.74241 | 2.52207 | 8.97875 | 12.7843 | 10.1525 | 27.9964 | 25.6519 | 25.4027 |
| OAHFA(22:5/18:0) | OAHFA | 0.03806 | 17.024 | 9.69464 | 13.9849 | 9.2917 | 31.7904 | 5.39154 | 1.28613 | 0.41483 | 4.6236 | 0.34851 | 0.17555 | 0.59745 |
| DG(16:0e) | DG | 0.05971 | 35.48 | 21.0284 | 26.7838 | 31.9445 | 12.2354 | 19.6172 | 2.59166 | 1.82501 | 11.3236 | 18.082 | 16.9787 | 13.3617 |
| OAHFA(18:2/33:1) | OAHFA | 0.11049 | 5.60142 | 6.53719 | 9.0959 | 6.08721 | 5.89605 | 4.29996 | 6.50532 | 10.0691 | 8.96796 | 35.3648 | 46.1287 | 28.8667 |
| Cer(d36:0) | Cer | 0.09705 | 22.7607 | 31.18 | 20.9294 | 29.0257 | 15.313 | 21.5531 | 31.1372 | 48.8511 | 21.562 | 38.318 | 59.2061 | 33.7215 |
| OAHFA(16:0/33:1) | OAHFA | 0.02326 | 2.54355 | 4.76193 | 7.65784 | 4.6034 | 5.01557 | 1.99912 | 7.70597 | 11.8403 | 10.5895 | 24.2012 | 23.7539 | 22.9009 |
| DG(32:2p) | DG | 0.00306 | 17.4053 | 6.30851 | 12.4845 | 7.13482 | 12.911 | 13.1642 | 0.84207 | 0.54142 | 1.49934 | 0.60449 | 0.41325 | 1.4242 |
| Cer(d18:1/22:0) | Cer | 0.00494 | 15.6019 | 11.3758 | 14.4966 | 11.0668 | 22.0587 | 10.494 | 1.84644 | 4.288 | 2.33799 | 4.31378 | 4.25014 | 5.53299 |
| SM(d42:3) | SM | 0.00636 | 7.68175 | 14.031 | 10.6404 | 8.43206 | 20.5675 | 10.6217 | 1.47427 | 3.53951 | 0.87806 | 1.82278 | 1.46981 | 1.16418 |
| OAHFA(18:0/35:1) | OAHFA | 0.02495 | 1.81721 | 4.08372 | 4.26764 | 3.90826 | 2.3288 | 1.47114 | 6.96398 | 10.3465 | 7.65208 | 25.2338 | 19.8585 | 19.8218 |
| Cer(d18:1/20:0+O) | Cer | 0.01483 | 12.5686 | 14.6836 | 27.5156 | 11.4481 | 19.5166 | 12.4802 | 3.44933 | 6.37698 | 5.8403 | 4.26676 | 5.60735 | 5.4027 |
| SM(d42:3)+CH3COO | SM | 0.0019 | 8.8498 | 10.3997 | 9.16558 | 9.24999 | 15.5708 | 9.0667 | 0.99033 | 2.35971 | 0.27557 | 2.03973 | 0.67644 | 0.54974 |
| PG(17:0/18:1) | PG | 0.04758 | 2.9871 | 0.55283 | 11.5824 | 2.50536 | 3.19627 | 1.06396 | 22.3214 | 23.0919 | 14.5303 | 3.50928 | 8.82946 | 18.9624 |
| SM(d40:0) | SM | 0.00333 | 6.46003 | 15.3362 | 9.8061 | 7.54021 | 14.4123 | 9.1375 | 0.34115 | 2.32091 | 0.79419 | 0.95884 | 1.64335 | 1.28452 |
| Cer(d18:1/16:1) | Cer | 0.06362 | 20.9198 | 37.1622 | 14.9287 | 24.1611 | 26.7978 | 18.0685 | 15.8296 | 16.235 | 12.8515 | 6.35872 | 6.70376 | 16.9514 |
| PC(17:1/15:0) | PC | 0.11679 | 0.40239 | 0.28639 | 0.72884 | 0.95831 | 0.0246 | 0.14768 | 32.2559 | 25.3616 | 19.1345 | 1.34963 | 1.80056 | 2.12119 |
| TG(34:0p) | TG | 0.02361 | 16.9032 | 8.84641 | 13.9188 | 5.61056 | 19.967 | 3.00712 | 0.87245 | 0.53653 | 2.51318 | 0.03018 | 0.07849 | 0.82327 |
| PE(35:1) | PE | 0.12787 | 0.40125 | 0.27922 | 0.67771 | 1.01615 | 0.02365 | 0.14532 | 34.3068 | 24.5426 | 17.9921 | 1.38941 | 1.66404 | 2.03054 |
| Cer(d18:1/24:1) | Cer | 0.00267 | 13.3402 | 9.63426 | 13.825 | 8.78559 | 16.8684 | 9.75791 | 2.13592 | 2.97329 | 3.7324 | 4.18321 | 1.78624 | 2.84694 |
| DG(39:6) | DG | 0.04931 | 34.4758 | 21.7535 | 20.9294 | 19.1343 | 13.3614 | 15.8745 | 8.4089 | 19.0724 | 8.15357 | 5.40594 | 5.95194 | 9.19184 |
| TG(14:0e/15:0/15:1) | TG | 0.04558 | 6.22573 | 2.62854 | 2.50275 | 1.96208 | 0.49063 | 4.42678 | 14.6363 | 19.2187 | 21.7048 | 4.84671 | 3.79044 | 22.481 |
| PE(15:0/18:1) | PE | 0.10533 | 8.33444 | 5.3296 | 10.4794 | 6.50242 | 4.06847 | 2.31019 | 24.9843 | 37.1503 | 19.7057 | 8.26426 | 4.54227 | 16.0631 |
| SM(d42:1)+CH3COO | SM | 0.00549 | 6.92933 | 9.82971 | 8.81448 | 9.17234 | 17.5172 | 8.40242 | 0.0026 | 1.72526 | 1.06357 | 3.44486 | 0.89145 | 1.21633 |
| Cer(d18:0/24:1) | Cer | 0.00379 | 8.02988 | 10.2585 | 11.2154 | 6.9334 | 15.8303 | 6.46724 | 0.71952 | 1.94751 | 0.86187 | 0.41566 | 1.84396 | 1.518 |
| PC(15:1/15:0) | PC | 0.11071 | 8.35118 | 5.83718 | 11.0794 | 7.02131 | 4.03093 | 2.50378 | 24.9843 | 37.1503 | 19.7057 | 8.26426 | 5.10614 | 15.3379 |
| PG(33:0p) | PG | 0.07935 | 3.43524 | 2.95722 | 6.47069 | 4.48196 | 3.9045 | 1.95526 | 23.5141 | 21.2363 | 26.2312 | 5.98918 | 4.32504 | 8.68557 |
| Cer(d18:0/24:0) | Cer | 0.13646 | 32.0862 | 26.6495 | 31.8013 | 27.6931 | 49.0481 | 21.3808 | 7.4166 | 19.8285 | 9.71959 | 22.0266 | 33.155 | 23.0934 |
| SM(d18:1/15:0) | SM | 0.02595 | 7.19641 | 13.5053 | 18.734 | 10.9617 | 21.9937 | 6.60791 | 2.18147 | 4.32932 | 3.41279 | 4.92956 | 2.56073 | 3.71662 |
| LPC(20:0e) | LPC | 0.00746 | 5.08769 | 15.3906 | 4.9616 | 8.67529 | 12.1604 | 8.75032 | 1.08098 | 0.7776 | 0.34634 | 0.66908 | 1.29588 | 0.89426 |
| PE(17:1/18:1) | PE | 0.11662 | 0.04069 | 0.06361 | 0.48561 | 0.25049 | 0.03345 | 0.20307 | 12.6066 | 15.5281 | 36.3739 | 2.05168 | 2.11304 | 7.08365 |
| LPC(18:0e) | LPC | 0.0599 | 6.19226 | 25.379 | 3.84926 | 9.46985 | 12.2354 | 9.79571 | 0.84372 | 2.3491 | 0.75511 | 1.54543 | 2.62612 | 1.93989 |
| DG(38:5p) | DG | 0.03546 | 5.5563 | 21.9347 | 4.30298 | 9.45364 | 8.93261 | 8.29861 | 0.55082 | 0.81913 | 1.28311 | 0.52266 | 0.579 | 0.91998 |
| PG(30:0p) | PG | 0.12787 | 1.21443 | 0.52671 | 4.64002 | 1.05419 | 1.45014 | 0.03802 | 34.5896 | 15.0525 | 15.5856 | 2.03406 | 4.98868 | 5.07504 |
| PE(15:0/20:4) | PE | 0.08533 | 7.05464 | 1.50229 | 4.26891 | 3.89659 | 7.0358 | 5.9926 | 27.2628 | 25.3598 | 6.99671 | 9.19005 | 10.9076 | 10.6105 |
| TG(18:0/16:0/18:2) | TG | 0.02441 | 12.9201 | 6.23599 | 9.26457 | 3.21067 | 18.6159 | 4.91722 | 0.09872 | 0.17384 | 0.73345 | 0.0304 | 0.01322 | 0.23518 |
| MG(38:6) | MG | 0.04052 | 5.18811 | 21.9347 | 4.30298 | 9.50228 | 8.93261 | 7.52424 | 0.55082 | 0.81913 | 1.28311 | 0.52266 | 0.579 | 0.91998 |
| OAHFA(20:4/18:0) | OAHFA | 0.02018 | 10.7799 | 22.2079 | 14.9596 | 12.1195 | 18.1659 | 22.4654 | 6.33927 | 8.51398 | 10.3877 | 7.22382 | 8.15217 | 8.43488 |
| PG(51:2) | PG | 0.05345 | 0.15604 | 0.68031 | 3.23549 | 1.10974 | 0.08514 | 0.06673 | 18.7432 | 14.3857 | 15.266 | 4.23317 | 2.09076 | 5.02254 |
| LPE(20:1p) | LPE | 0.08838 | 7.45669 | 18.7693 | 1.44515 | 5.85991 | 11.1672 | 26.0288 | 1.1931 | 1.68304 | 0.63967 | 2.64996 | 0.82907 | 3.76813 |
| Cer(d18:1/20:0) | Cer | 0.00306 | 11.3568 | 6.76951 | 11.2491 | 11.5645 | 13.3649 | 8.92628 | 2.1934 | 4.76856 | 2.75924 | 1.569 | 2.27269 | 5.82285 |
| Cer(d18:2/20:0+O) | Cer | 0.00549 | 9.99129 | 7.05175 | 12.0893 | 7.55642 | 12.7609 | 4.34935 | 1.92044 | 1.38716 | 2.94157 | 1.33898 | 1.16491 | 1.3935 |
| phSM(d34:0) | phSM | 0.09205 | 10.2925 | 16.8589 | 7.72779 | 23.6746 | 23.7953 | 26.8446 | 3.43068 | 8.89266 | 5.98309 | 12.5103 | 6.57846 | 14.5764 |
| OAHFA(21:0/34:1) | OAHFA | 0.0257 | 1.07656 | 2.15366 | 1.99356 | 2.45526 | 1.19543 | 0.99378 | 4.14152 | 7.89606 | 5.42105 | 18.9068 | 11.6614 | 12.0036 |
| PG(20:1p/16:0) | PG | 0.11057 | 0.20713 | 0.73168 | 4.83703 | 0.98253 | 0.82491 | 0.39131 | 20.4471 | 7.29914 | 25.7066 | 2.40932 | 8.27907 | 2.46972 |
| SM(d42:1) | SM | 0.02038 | 4.40152 | 13.3784 | 8.62059 | 6.14567 | 14.6375 | 5.66577 | 0.35735 | 3.74428 | 0.82719 | 1.32781 | 1.41267 | 1.22917 |
| PG(18:0p/15:0) | PG | 0.10697 | 2.84185 | 1.04039 | 6.56267 | 2.81187 | 2.93782 | 0.16088 | 22.6622 | 16.1687 | 21.5096 | 2.70291 | 4.85675 | 4.69193 |
| dMePE(33:2) | dMePE | 0.09781 | 5.35314 | 0.04338 | 3.03908 | 0.09003 | 1.23787 | 2.09769 | 23.8549 | 12.481 | 14.7138 | 4.07735 | 2.25928 | 6.43301 |
| PI(54:7) | PI | 0.01392 | 1.9971 | 4.93272 | 1.97377 | 0.14268 | 0.43733 | 2.96562 | 12.2899 | 4.38876 | 5.07157 | 10.4595 | 10.4133 | 11.3509 |
| OAHFA(38:2) | OAHFA | 0.02694 | 9.45618 | 17.7663 | 18.0366 | 19.0246 | 13.6245 | 13.3865 | 4.45646 | 6.31025 | 5.47553 | 11.9556 | 10.5741 | 9.16157 |
| LPE(18:2p) | LPE | 0.13646 | 12.0528 | 24.2138 | 1.69427 | 7.32024 | 7.41854 | 25.564 | 4.77161 | 2.28208 | 2.62914 | 3.27337 | 0.32654 | 9.05906 |
| TG(40:4) | TG | 0.08793 | 11.9996 | 11.0761 | 10.7428 | 11.4806 | 5.20193 | 9.73118 | 14.3194 | 23.431 | 10.1813 | 16.8392 | 31.138 | 16.0449 |
| TG(16:0p/9:0/9:0) | TG | 0.01706 | 11.8992 | 4.13316 | 7.09845 | 3.14581 | 12.3105 | 5.3173 | 0.22705 | 0.55956 | 1.41573 | 0.00697 | 0.2733 | 0.17453 |
| DG(34:1) | DG | 0.01706 | 11.8992 | 4.13316 | 7.09845 | 3.14581 | 12.3105 | 5.3173 | 0.22705 | 0.55956 | 1.41573 | 0.00697 | 0.2733 | 0.17453 |
| Cer(d18:2/24:1) | Cer | 0.05027 | 10.0415 | 18.6717 | 13.5822 | 10.7509 | 13.0611 | 16.2617 | 2.03231 | 6.34772 | 2.05624 | 3.5004 | 13.3762 | 10.4247 |
| TG(16:0e/15:0/17:1) | TG | 0.12894 | 10.9118 | 4.80389 | 3.67363 | 3.64849 | 0.57271 | 6.23364 | 13.3871 | 16.118 | 19.4201 | 5.50951 | 2.97012 | 28.6452 |
| SM(d24:0/16:0)+CH3COO | SM | 0.00333 | 3.84598 | 8.58682 | 8.15397 | 8.27229 | 3.21796 | 7.30998 | 1.2E-05 | 1.33665 | 0.01043 | 0.14735 | 0.51946 | 0.63591 |
| CerG1(d34:0+O) | CerG1 | 0.02263 | 9.20471 | 6.19974 | 6.33738 | 4.9133 | 4.20358 | 6.50466 | 18.2721 | 14.3628 | 14.2366 | 11.1433 | 7.07967 | 11.8932 |
| PEt(31:1e) | PEt | 0.02206 | 4.88686 | 6.25412 | 3.498 | 3.43769 | 14.3372 | 8.0792 | 0.14049 | 0.07047 | 0.32318 | 0.05201 | 0.04592 | 0.08909 |
| OAHFA(18:2/34:2) | OAHFA | 0.12894 | 3.90608 | 4.27666 | 6.98689 | 8.05514 | 8.22148 | 4.20035 | 4.67697 | 10.3435 | 7.1625 | 23.5616 | 29.407 | 16.1089 |
| OAHFA(22:0/34:1) | OAHFA | 0.01473 | 1.37443 | 2.68831 | 2.85578 | 2.392 | 2.21775 | 1.01803 | 4.07012 | 9.45984 | 5.45502 | 13.6804 | 7.98411 | 11.2303 |
| LPE(18:0e) | LPE | 0.00423 | 4.65256 | 7.9944 | 3.24919 | 9.06446 | 7.95678 | 5.12371 | 0.32666 | 0.78953 | 0.22764 | 1.36888 | 1.21798 | 0.8077 |
| PG(32:1p) | PG | 0.03418 | 0.58533 | 1.68331 | 4.23203 | 1.93909 | 0.51475 | 0.18525 | 14.4314 | 9.55501 | 11.0485 | 3.16198 | 3.79179 | 6.86318 |
| OAHFA(18:0/18:0) | OAHFA | 0.07096 | 9.67691 | 5.12807 | 4.76084 | 3.54812 | 19.0742 | 3.65671 | 0.54997 | 0.27654 | 1.47408 | 0.27244 | 0.52369 | 0.01067 |
| DG(36:1p) | DG | 0.04472 | 4.83666 | 7.75874 | 11.138 | 3.71335 | 15.8385 | 3.30396 | 0.66579 | 1.84119 | 1.94201 | 0.66916 | 0.86306 | 0.68367 |
| DG(36:2p) | DG | 0.0177 | 7.04578 | 7.90376 | 13.3919 | 3.12959 | 7.35627 | 5.47218 | 1.05305 | 0.81701 | 3.88401 | 0.06122 | 0.14399 | 0.57334 |
| SM(d41:1)+CH3COO | SM | 0.00286 | 5.12786 | 7.08036 | 5.79504 | 4.33688 | 9.3821 | 4.62936 | 0.15484 | 0.37494 | 1.60272 | 1.4966 | 0.53964 | 0.88404 |
| Cer(d17:0/16:0) | Cer | 0.02873 | 12.9535 | 13.9947 | 7.9327 | 9.84281 | 3.13017 | 7.89852 | 3.28152 | 4.7096 | 4.1696 | 2.00302 | 1.28302 | 3.31776 |
| PC(32:0e) | PC | 0.10308 | 1.85768 | 6.50791 | 5.57631 | 7.65372 | 2.04174 | 1.84557 | 5.01551 | 20.0377 | 3.49847 | 17.0463 | 17.5112 | 8.62981 |
| Cer(d15:0/26:0) | Cer | 0.00293 | 7.94999 | 7.37581 | 7.89515 | 6.56471 | 10.9416 | 6.50415 | 1.17411 | 2.91098 | 1.99935 | 3.22274 | 4.00383 | 3.65466 |
| TG(6:0/10:0/18:2) | TG | 0.00367 | 9.35533 | 5.74654 | 9.77683 | 7.89695 | 5.23946 | 6.18201 | 1.03739 | 1.00402 | 2.48463 | 2.98259 | 1.37043 | 3.35402 |
| OAHFA(18:2/38:1) | OAHFA | 0.02441 | 1.12304 | 2.13224 | 3.30076 | 1.87408 | 1.83954 | 0.75633 | 3.36428 | 7.67304 | 3.82196 | 10.4666 | 13.1083 | 9.13906 |
| PC(36:4p) | PC | 0.06021 | 17.4053 | 7.68622 | 9.55729 | 10.6049 | 8.10691 | 2.49087 | 2.6103 | 4.76811 | 4.15532 | 2.44407 | 2.47613 | 2.37501 |
| SM(d32:1) | SM | 0.0051 | 4.36805 | 7.39618 | 7.15699 | 3.48633 | 8.40716 | 3.87182 | 0.8279 | 1.24234 | 1.27117 | 1.05851 | 0.40844 | 0.90069 |
| Cer(d38:0) | Cer | 0.09333 | 10.6607 | 14.1035 | 9.51338 | 13.0535 | 7.21365 | 9.49887 | 14.1329 | 22.6704 | 9.69575 | 16.8392 | 25.9066 | 15.7005 |
| TG(4:0/13:0/17:3) | TG | 0.01229 | 6.74454 | 4.65887 | 10.0988 | 8.20504 | 2.38703 | 5.44636 | 0.39075 | 0.28566 | 1.12908 | 1.34085 | 0.25459 | 2.33875 |
| CerG1(d18:0/16:0+O) | CerG1 | 0.02476 | 9.60637 | 5.45649 | 9.82074 | 3.8755 | 8.18197 | 4.94303 | 14.3194 | 14.0118 | 14.1224 | 10.8326 | 10.5882 | 9.35501 |
| PE(44:0) | PE | 0.00036 | 6.00816 | 4.73138 | 4.84451 | 4.58899 | 5.65982 | 3.98798 | 0.76369 | 1.4681 | 0.88383 | 0.28359 | 0.78229 | 0.43308 |
| SM(d18:0/23:1) | SM | 0.00286 | 4.08354 | 7.84938 | 4.71278 | 5.52949 | 7.22866 | 4.74944 | 0.70614 | 2.1441 | 2.14192 | 0.98044 | 1.0025 | 1.2359 |
| Cer(d18:1/18:0+O) | Cer | 0.00575 | 6.4935 | 9.49901 | 8.02051 | 5.88623 | 8.25703 | 6.04004 | 1.38734 | 5.49941 | 2.0848 | 2.8376 | 3.35188 | 1.68678 |
| TG(6:0/12:0/16:2) | TG | 0.01329 | 8.66916 | 5.49275 | 9.26457 | 8.38341 | 2.342 | 5.76902 | 0.97653 | 0.43371 | 1.87061 | 2.85832 | 1.39177 | 1.90364 |
| SM(d37:3) | SM | 0.00494 | 6.61065 | 5.69216 | 7.21554 | 6.94023 | 3.5205 | 4.47841 | 0.34277 | 0.65119 | 3.84117 | 1.34961 | 0.15603 | 0.4472 |
| PE(37:3p) | PE | 0.07245 | 8.46833 | 5.71028 | 5.63485 | 4.62142 | 4.36122 | 7.09834 | 11.6158 | 14.7431 | 19.7057 | 7.80859 | 6.57846 | 10.8417 |
| PE(36:2e) | PE | 0.11057 | 9.23818 | 8.01253 | 7.55216 | 9.77795 | 2.99505 | 7.39518 | 10.1429 | 16.0009 | 7.63951 | 10.8326 | 24.8728 | 14.5764 |
| Cer(d18:2/18:2) | Cer | 0.00333 | 2.4267 | 4.35069 | 7.42044 | 5.22139 | 6.56059 | 4.63328 | 0.28606 | 0.34322 | 1.00091 | 0.91944 | 0.87148 | 0.45228 |
| SM(d42:0)+CH3COO | SM | 0.0625 | 0.10554 | 8.80621 | 5.49616 | 7.08624 | 10.0713 | 1.04134 | 0.12275 | 0.73294 | 0.12526 | 0.16899 | 0.90876 | 0.44008 |
| Cer(d44:4) | Cer | 0.00292 | 3.17981 | 7.25116 | 4.97623 | 4.32954 | 6.05015 | 5.7432 | 0.57361 | 1.99688 | 0.69083 | 0.92825 | 1.85058 | 1.38374 |
| Cer(d18:1/18:2) | Cer | 0.02753 | 9.45574 | 10.2966 | 11.9283 | 9.12933 | 6.82331 | 7.73074 | 2.01366 | 5.11913 | 3.08436 | 2.6719 | 9.24117 | 4.44182 |
