# Supplementary material for: The gut microbiota contributes to changes in the host immune response induced by Trichinella spiralis
Source: PLoS Negl Trop Dis. 2023 Aug 16;17(8):e0011479. doi: 10.1371/journal.pntd.0011479 (PMC10431649; doi:10.1371/journal.pntd.0011479)
Supplement: S1 Code — (DOCX) [file pntd.0011479.s011.docx]

**S1 Code:** **The analysis and plotting code in Fig 1.**

**####figure1**

library(ggplot2)

library(dplyr)

library(vegan)

library(ggpubr)

library(reshape2)

library (ComplexHeatmap)

library(circlize)

map <- read.table("map.txt",header = T,sep = "\t")

dt <- read.table("unweighted_unifrac_otu_table_even.txt",header = T,sep = "\t",row.names = 1)

A# pcoa

raw_pcoa <- cmdscale(dt, k = 10)

temp <- as.data.frame(raw_pcoa[,1:2])

pc_importance <- round(raw_pcoa$eig/sum(raw_pcoa$eig)*100,digits = 2)

plot_data <- merge(temp, map, by.x = "row.names",by.y = "ID")

mycol <- c("#b3e2cd","#fdcdac","#cbd5e8","#f4cae4","#e6f5c9","#fff2ae")

plot_data$V4 <- plot_data$V2*-1

ggplot(plot_data, aes(x=V1, y=V4, fill=Group))+

stat_ellipse(level = 0.8, show.legend = T) +

geom_point(colour = "black", size = 4, shape = 21, stroke = 1) +

labs(x=paste("PCoA 1 (", pc_importance[1],digits=4,"%)", sep=""),

y=paste("PCoA 2 (", pc_importance[2],digits=4, "%)", sep=""),

title="PCoA")+

geom_text(aes(V1, V4, label = Row.names))+

scale_fill_manual(values = mycol)+

theme_bw()

B# shannon index boxplot

shannon <- read.table("shannon.txt",header = T, sep = "\t")

shannon.m <- merge(shannon, map, by.x = "Samples", by.y = "ID")

box_plot(shannon.m, Group = "Group", Value = "shannon")

sig_list <- t(combn(unique(shannon.m$Group),2))

compire<-list()

for(i in 1:nrow(sig_list)){

compire[[i]]<-as.character(sig_list[i,])

}

shannon.m$Group <- factor(shannon.m$Group,

levels = c("CK","Ts3","Ts8","Ts17","Ts40","CK40"))

ggplot(shannon.m, aes(x = Group, y = shannon))+

geom_boxplot(aes(fill = Group))+

scale_fill_manual(values = mycol)+

# geom_signif(comparisons = compire, step_increase=0.1,

# map_signif_level = T, test = wilcox.test)+

labs(title="", x="", y = "", fill = "")+

theme_bw()

C# barplot

x1 <- data.frame(ID= paste("PC",1:35,sep = ""),PC = pc_importance)

x2 <- head(x1,10)

x2$ID <- factor (x2$ID, levels = x2$ID)

ggplot(data=x2, aes(x = ID, y = PC))+

geom_bar(stat = "identity",position = "identity")+

scale_fill_manual(values = c("purple", "blue"), guide=FALSE)+

xlab("")+ ylab("")+

geom_text(aes(y = PC-1, label = PC ))+

theme_bw()

D# genus barplot

genus <- read.table("genus15.txt",header = T, sep = "\t",row.names = 1)

genus.t <- as.data.frame(t(genus))

genus.m <- merge (genus.t, map, by.x = "row.names", by.y = "ID")

genus.melt <- melt(genus.m)

genus.melt$variable <- factor(genus.melt$variable,levels = unique(genus.melt$variable))

mycols2 <- c("#a6cee3","#1f78b4","#b2df8a","#33a02c",

"#fb9a99","#e31a1c","#fdbf6f","#ff7f00",

"#cab2d6","#6a3d9a","#ffff99","#b15928",

"#fdcdac","#cbd5e8","#e6f5c9","#fff2ae")

genus.melt$Row.names <- as.character(genus.melt$Row.names)

ggplot(genus.melt, aes(x = Row.names, y = value, fill = variable))+

geom_bar(stat = 'identity', position = 'stack', width = 1)+

scale_fill_manual(values = mycols2)+

facet_wrap(~Group,scales = "free")

E# heatmap

meta1 <- as.matrix(read.table("meta.txt", sep = "\t",header = T, row.names = 1))

meta2 <- t(scale(t(meta1), center = TRUE, scale = TRUE))

m1 <- read.table("m1.txt", sep = "\t",header = T)

m2 <- read.table("m2.txt", sep = "\t",header = T)

heat.map <- function(matrix, p.matrix = NA, m1, m2){

ht <- heatmap(matrix)

row_order <- rownames(matrix)[ht$rowInd]

col_order <- colnames(matrix)[ht$colInd]

matrix <- matrix[row_order, col_order]

rownames(m1) <- m1[,1]

m1 <- m1[colnames(matrix),]

rownames(m2) <- m2[,1]

m2 <- m2[rownames(matrix),]

csd <- data.frame(enriched = m1[,2])

csd$enriched <- factor(csd$enriched, unique(csd$enriched))

rsd <- data.frame(enriched = m2[,2])

rsd$enriched <- factor(rsd$enriched, unique(rsd$enriched))

mycols = colorRamp2(breaks=c(-2,0, 2),

colors=c("#2c7bb6","#f7f7f7", "#d7191c"))

Heatmap(as.matrix(matrix),#bottom_annotation = ha2,

cluster_rows=FALSE, cluster_columns=FALSE,

row_split=rsd, column_split=csd,

row_title_side='left',column_title_side = 'top',

row_title_rot = 0, column_title_rot = 90,

border=T,

show_row_names=T, show_column_names = T,

row_gap = unit(0, 'mm'), column_gap = unit(0, 'mm'),

col = mycols

)

}

heat.map (meta2, m1 = m2, m2 = m1)
